# *orsai*, the Drosophila homolog of human ETFRF1, links lipid catabolism to growth control

**DOI:** 10.1186/s12915-022-01417-w

**Published:** 2022-10-21

**Authors:** Magdalena Fernandez-Acosta, Juan I. Romero, Guillermo Bernabó, Giovanna M. Velázquez-Campos, Nerina Gonzalez, M. Lucía Mares, Santiago Werbajh, L. Amaranta Avendaño-Vázquez, Gerald N. Rechberger, Ronald P. Kühnlein, Cristina Marino-Buslje, Rafael Cantera, Carolina Rezaval, M. Fernanda Ceriani

**Affiliations:** 1 Laboratorio de Genética del Comportamiento, Fundación Instituto Leloir – Instituto de Investigaciones Bioquímicas de Buenos Aires (IIBBA- CONICET), Buenos Aires, Argentina; 2 Present Address: Innovid, Buenos Aires, Argentina; 3 Present Address: Fundación Cassará, Buenos Aires, Argentina; 4 Present Address: IFIBYNE-CONICET, Buenos Aires, Argentina; 5 Institute for Molecular Biosciences, University of Graz, Graz, Austria; 6 BioTechMed-Graz, Graz, Austria; 7 Field of Excellence BioHealth – University of Graz, Graz, Austria; 8 Laboratorio de Bioinformática Estructural, Fundación Instituto Leloir – Instituto de Investigaciones Bioquímicas de Buenos Aires (IIBBA- CONICET), Buenos Aires, Argentina; 9 Departamento de Biología del Neurodesarrollo, Instituto de Investigaciones Biológicas Clemente Estable, Montevideo, Uruguay; 10 Zoology Department, Stockholm University, Stockholm, Sweden; 11 Present Address: School of Biosciences, University of Birmingham, Birmingham, UK

**Keywords:** CG6115, LYR, ETFRF1, Fat body, Lipid metabolism, Lipid droplets, *Drosophila melanogaster*

## Abstract

**Background:**

Lipid homeostasis is an evolutionarily conserved process that is crucial for energy production, storage and consumption. *Drosophila* larvae feed continuously to achieve the roughly 200-fold increase in size and accumulate sufficient reserves to provide all energy and nutrients necessary for the development of the adult fly. The mechanisms controlling this metabolic program are poorly understood.

**Results:**

Herein we identified a highly conserved gene, *orsai* (*osi*), as a key player in lipid metabolism in *Drosophila*. Lack of *osi* function in the larval fat body, the regulatory hub of lipid homeostasis, reduces lipid reserves and energy output, evidenced by decreased ATP production and increased ROS levels. Metabolic defects due to reduced Orsai (Osi) in time trigger defective food-seeking behavior and lethality*.* Further, we demonstrate that downregulation of *Lipase* 3, a fat body-specific lipase involved in lipid catabolism in response to starvation, rescues the reduced lipid droplet size associated with defective *orsai*. Finally, we show that *osi*-related phenotypes are rescued through the expression of its human ortholog ETFRF1/LYRm5, known to modulate the entry of β-oxidation products into the electron transport chain; moreover, knocking down electron transport flavoproteins EtfQ0 and *walrus*/ETFA rescues *osi*-related phenotypes, further supporting this mode of action.

**Conclusions:**

These findings suggest that Osi may act in concert with the ETF complex to coordinate lipid homeostasis in the fat body in response to stage-specific demands, supporting cellular functions that in turn result in an adaptive behavioral response.

**Supplementary Information:**

The online version contains supplementary material available at 10.1186/s12915-022-01417-w.

## Background

A balanced interplay between different metabolic pathways is key to cellular homeostasis and ultimately to the survival of the organism. A fundamental aspect of this balance is the coordination between carbohydrate and lipid metabolism [1].

Abnormal lipid metabolism could result either from the inability to properly metabolize lipids when they are needed to sustain cell homeostasis (i.e., as a result of a deficient enzymatic function [2, 3]) or could result from an overuse of lipid reserves even in the presence of more readily available energy sources [4]. In humans, lipid metabolism disorders span a broad spectrum of conditions, from hypercholesterolemia and hypertriglyceridemia [5] to those where the cell is incapable of breaking down lipids such as Tay-Sachs and Gaucher diseases; in fact, there are up to 22 fatty acid oxidation disorders caused either by disruption of mitochondrial β-oxidation or the transport of fatty acids through the carnitine transporter [6, 7]. Genetic conditions that lead to a deficient regulation of β-oxidation may cause hypotonia, myopathies, neuropathies, organ failure, and even developmental delay, intolerance to fasting, and death [8].

*Drosophila* has organ systems that perform essentially the same metabolic functions as their vertebrate counterparts [9]. For example, there are oxidative and glycolytic muscles. In addition, the fat body, which stores excess lipids in the form of triglycerides, combines functions of the liver and the white adipose tissue. Lipids are stored as lipid droplets that can be mobilized in times of need using lipases that are orthologous to those found in mammals [9, 10].

During development, the *Drosophila* embryo undergoes a metabolic switch from oxidative phosphorylation to aerobic glycolysis, where the synthesis of amino acids and nucleotides is promoted [11]. A characteristic of this carbohydrate-dependent developmental stage is the uncoupling of β-oxidation to promote the synthesis of lipids that will be used as building blocks to support membrane homeostasis and cell growth [12]. This transition allows larvae to generate sufficient biomass to support the nearly 200-fold increase in size associated with larval development [13, 14]. Such energy reserves are mainly stored in lipid droplets within the fat body and under normal conditions would not be used until the onset of metamorphosis.

The inability to exploit an energy resource due to metabolic dysfunction not only correlates with an energy-depleted state. It may also preclude the use of specific nutrients which causes aberrant behaviors such as hyperphagia [15], a change of diet choice [16], or early wandering [17], and even the inability to live on certain diets or survive starvation [18–20].

In the present study, we investigated the function of a novel *Drosophila* gene involved in lipid metabolism that we named *orsai* (*osi*). The lack of *osi* in larvae causes an overactive lipid catabolism, which impacts cellular dynamics and feeding behavior, ultimately leading to death in early stages of development. We show that Orsai has a critical role in the regulation of β-oxidation and provide evidence that it is an ortholog of human ETFRF1/LYRm5. Delving into Orsai’s function sheds light not only on the control of lipid metabolism and its relationship with growth promotion but also on the link between metabolism and behavior.

## Results

### Identification of a novel mutant displaying stunted growth and developmental arrest

**Fig. 1:**
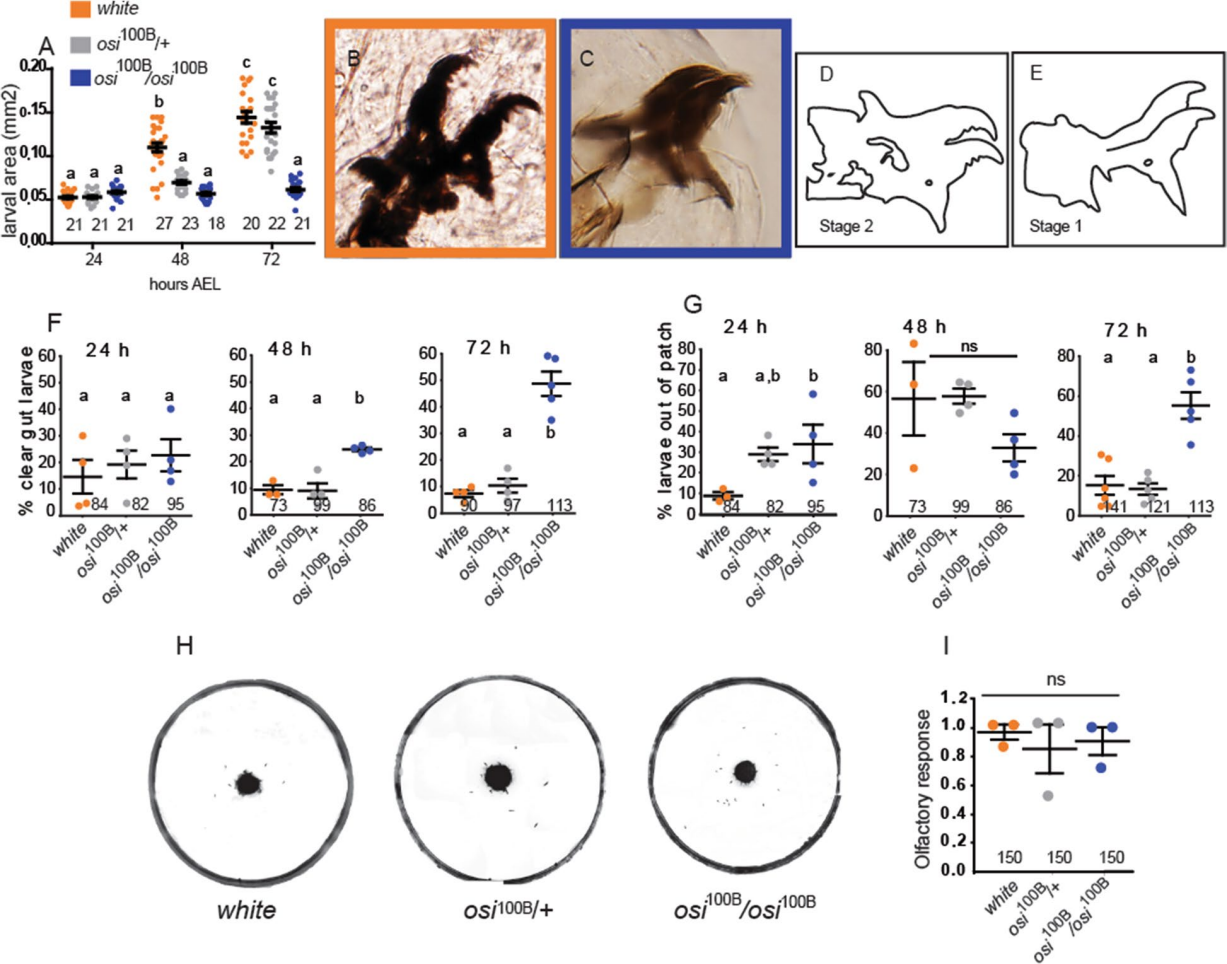
A P[UAS] insertional mutant exhibits abnormal food-seeking behavior. The P[UAS]^100B^ insertion triggers developmental arrest and premature death. **A** Graph shows the size of larvae grown in standard agar plates at 24, 48, and 72h AEL. Homozygous P[UAS]^100B^ larvae barely increase their initial size at hatching and die around 72–80h AEL without any of the morphological correlates of third instar. Individuals were taken from 3 independent agar plates. **B–E** *osi* mutants die at first instar (L1). **B, C** Images correspond to mouth hooks from a control second instar (L2, **B**) and an *osi* mutant larva 72h AEL (**C**). **D, E** Schematic drawings of mouth hooks from a L2, showing three median teeth (**D**) and a L1, displaying a single tooth in the middle of the hook (**E**). **F** Proportion of animals registered as clear gut larvae representing those that did not feed during the experiment (control, P[UAS]^100B+/−^ and P[UAS]^100B^). **G** Percentage of the population outside of the yeast patch, highlighting the aberrant mutant behavior. In **F** and **G**, each dot represents an independent determination comprising an initial number of 25 larvae per genotype. **H** Representative images of 72h larvae in agar plates supplemented with a colored yeast patch (control, heterozygous and homozygous P[UAS]^100B^). Homozygous P[UAS]^100B^ insertion associates with developmental arrest. **I** Olfactory responses are spared in mutant larvae, showing no differences between genotypes. Each dot represents the collected olfactory response of 50 larvae. All panels: The total number of animals is expressed in the graph under each data set. Shapiro-Wilk test was used for normality assessment, when normality was confirmed one-way ANOVA with Bonferroni’s multiple comparisons was performed. For nonparametric assessment, Kruskal-Wallis with Dunn’s multiple comparisons test were performed (see Additional file 2). Different letters indicate significant differences, *p*<0.05; treatments sharing any letter are not statistically different. The total number of samples analyzed is included in the corresponding column (bottom). All datasets and statistical analysis on which the conclusions are based are included in Additional file 2

A number of years ago, we carried out a genetic screen to identify genes associated with cellular homeostasis [21]. One of the identified insertions (P[UAS]^100B^), as homozygotes, resulted in small and translucent larvae, which apparently die as first instar (L1). To better understand the impact of this mutation, the size of carefully staged larvae grown on standard agar plates (3% sucrose and a yeast patch mixed with food colorant) was examined at 24, 48, and 72 h after egg laying (AEL, Fig. 1). Three-day-old homozygous P[UAS]^100B^ mutants barely increased their initial size, while heterozygous ones appeared to catch up after an initial delay (Fig. 1A). In fact, homozygous P[UAS]^100B^ larvae did not molt and died around 72–80h AEL still exhibiting mouth hooks with the morphology of a wild-type L1 (Fig. 1B–E). These results strongly suggest that the mutation causes developmental arrest followed by premature death.

A closer inspection at the fair/translucent P[UAS]^100B^ homozygotes suggested abnormal feeding behavior. To begin to dissect the origin of this phenotype, we characterized homozygous mutant larvae in more detail. Food intake was assessed by monitoring the presence of colored food inside the gut (Fig. 1F). While most control animals (90%) had blue-colored gut within the hour that the experiment lasted, about 50% of P[UAS]^100B^ mutants showed a clear one. Notwithstanding, mutant larvae were able to ingest food (Fig. 1F), suggesting that no gross structural defects in feeding structures is responsible for the altered behavior.

Wild-type larvae feed continuously and tend to remain inside or near appetitive food. This characteristic behavior is easily quantified by assessing the number of larvae present in a yeast patch placed at the center of an agar plate. In this context, P[UAS]^100B^ homozygotes showed a weak preference to stay in the food during the first 48h AEL, shifting to no preference whatsoever at later time points (Fig. 1G, H). While only a small amount (<20%) of control larvae were found outside the food, over 50% of homozygous mutants did so around 24h AEL. To explore whether abnormal feeding derived from the inability to sense the food source, we performed a two-choice olfactory assay [22] that indicated that P[UAS]^100B^ mutants were equally capable of responding to an attractive cue present in the food as control larvae (Fig. 1I), ruling out the possibility that a generally defective sensory response could be the origin of the abnormal food-seeking behavior.

Considering the tendency of the mutant to be outside of the expected place at any given time, we named this mutant *orsai* (*osi*, Argentinian street language for the football term “offside”).

### The P[UAS]^100B^ insertion reduces the levels of endogenous CG6115

**Fig. 2:**
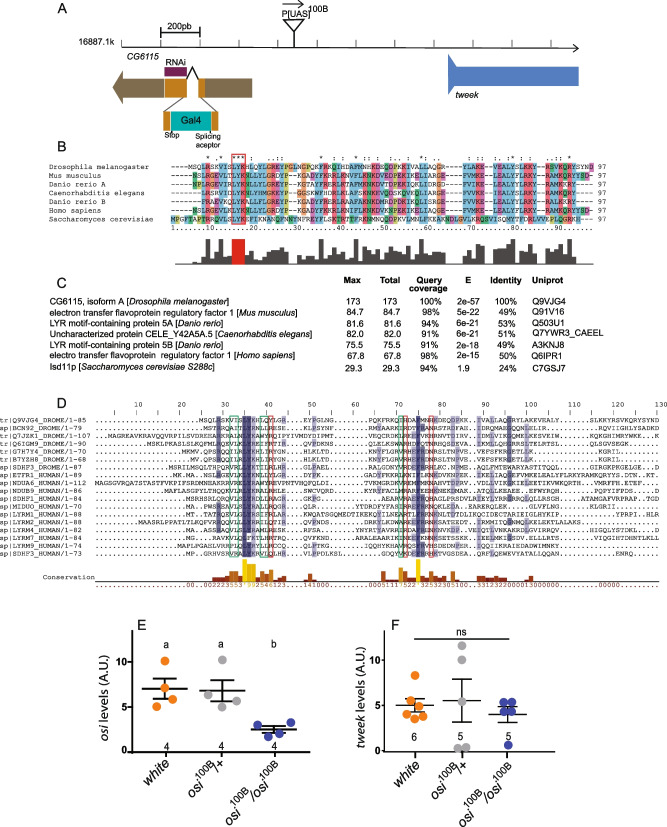
P[UAS]^100B^ is inserted in CG6115 and encodes a Complex I LYR domain-containing protein. **A** Schematic representation of the insertion locus for P[UAS]^100B^ and its nearest gene (*tweek*), indicating the target site of *osi*^RNAi^ in relation to the ORF in its mRNA, and the insertion site of the CRIMIC cassette containing Gal4 characteristic of *osi*^Gal4^. **B** Sequence analysis retrieved a series of different *osi* orthologs containing a Complex I_ LYR motif. **C** *osi* displays high levels of identity and similarity with genes present in the most common model species. **D** Multiple sequence alignment colored by conservation. Conserved hydrophobic residues are marked in green; conserved positive residues are marked in red. Bottom: histogram representing conserved residue frequencies. This image was generated by Jalview2 (doi:10.1093/bioinformatics/btp033). **E** qRT-PCR analysis at 72h shows that homozygosity for the P[UAS]^100B^ insertion reduced *osi* mRNA levels to about one third relative to control or heterozygous larvae. **F** qRT-PCR analysis at 72h AEL shows that homozygous P[UAS]^100B^ insertion did not affect *tweek* mRNA levels. For experiments shown in **E** and **F**, the number of independent observations is included under each data set; each replica included 30 larvae pooled together. A Shapiro-Wilk test was used for normality assessment, and when normality was confirmed, one-way ANOVA with Bonferroni’s multiple comparisons was performed. For nonparametric assessment, Kruskal-Wallis with Dunn’s multiple comparisons test was performed (see Additional file 2). Different letters indicate significant differences, *p*<0.05; treatments sharing any letter are not statistically different. The total number of analyzed samples is included in the corresponding panel (bottom). All datasets and statistical analysis on which the conclusions are based are included in Additional file 2

Plasmid rescue analysis [23] revealed that P[UAS]^100B^ is inserted 218 base pairs upstream of the transcriptional start site of *CG6115* (*osi*), and it is also upstream of the start site of *tweek* (Fig. 2A), but it does not physically interrupt any splice variant so far described for either gene. The P element is located in reverse orientation with regard to transcription at the *CG6115*. *osi* encodes a protein of 85 amino acids containing a Complex I_LYR motif (LYRm) according to Flybase (http://flybase.org/reports/FBgn0040985.html). The highly conserved tripeptide motif “LYR” is LYK in *Drosophila*; downstream, another highly conserved residue (a phenylalanine) is also present in Osi [24]. No additional motifs were identified. Sequence analysis retrieved putative orthologs in metazoans (Fig. 2B). Remarkably, Osi displays 50% identity and 76% similarity to the human LYRm5, whose molecular function has been examined and it has now been renamed Electron Transfer Flavoprotein Regulatory Factor 1 (ETFRF1 [25], Fig. 2C). In *Drosophila*, no functional characterization of CI_LYRm-containing proteins has been reported. Sequence analysis identified 7 entries in the fly genome according to Uniprot (CG6115, CG7712, CG42372, CG13191, CG34229, CG42372, CG3717). To explore the conservation within the Complex I_LYR family, *Drosophila* and human sequences were retrieved. The resulting set included 11 reviewed human as well as the 7 fly proteins (Fig. 2D). LYRm domain-containing proteins are characterized by the LYR/K motif. However, it is worth mentioning that their similarity extends beyond those 3 amino acids, showing two blocks of conserved residues. This is remarkable, particularly taking into account the divergence of this group of proteins (with an average of 25% identity). There is a striking conservation of four hydrophobic and one positive residue nearby the LYR/K motif, along with an absolutely conserved phenylalanine as well as one hydrophobic and two positively charged amino acids within the second block. This analysis suggests that a functional LYR/K domain likely contains those additional residues.

**Fig. 3:**
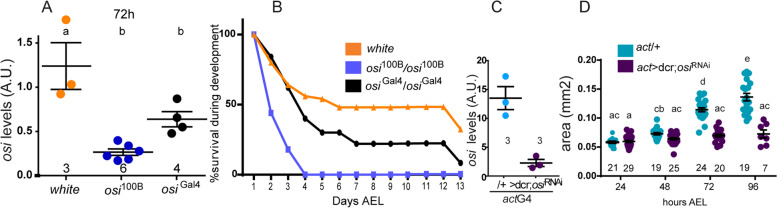
A reduction in *osi* levels affects viability. **A** Animals carrying a Gal4 insertion interrupting *osi* produce around 50% of *osi* levels compared to controls as confirmed by qPCR. For experiments shown in **E** and **F**, the number of independent observations is included under each data set; each replica included 30 larvae pooled together. One-way ANOVA was used to analyze statistical significance. **B** Homozygous *osi*^Gal4^ exhibit a poor performance during larval development, and only around 2% reach adulthood. Animals were counted every 24h until eclosion of controls. A representative experiment (out of two performed) is shown. **C** qPCR shows that expression of *osi*^RNAi^ in an ubiquitous pattern (*act*G4) decreases *osi* levels to about 20% of controls. The number of independent observations is included under each data set; each replica included 30 larvae pooled together. A two-tailed *T*-test was used to analyze statistical significance. **D** Downregulation of *osi* (*act*G4) produces a strong phenotype reminiscent of that of homozygous *osi*^100B^. Animals were measured every 24h until the death of all experimental individuals. *osi* downregulation stunt growth compared to controls and die between 72 and 96h. Animals were collected from two independent plates, and the number of independent observations is included under each data set. Two-way ANOVA was used to analyze statistical significance. All panels: Shapiro-Wilk test was used for normality assessment (see Additional file 2). Different letters indicate significant differences, *p*<0.05; treatments sharing any letter are not statistically different. The total number of samples analyzed is mentioned below each column. All datasets and statistical analysis on which the conclusions are based are included in Additional file 2

Quantitative real-time RT-PCR (qPCR) analysis indicated that, while heterozygous P[UAS]^100B^ did not affect *osi* mRNA levels, the homozygous one reduced them to about one third of the controls (Fig. 2E), without affecting *tweek* (Fig. 2F), which is located immediately downstream of the insertion (Fig. 2A), suggesting that reduced *osi* mRNA levels are responsible for the phenotype of the homozygous mutant larvae. We characterized a new insertion in CG6115 that we renamed *osi*^Gal4^. It contains a Gal4 gene trap that landed in the first and only coding intron; this cassette was designed so that it truncates the encoded protein [26]. qPCR analysis indicated that the insertion reduces *osi* levels to 50% compared to wild-type controls (Fig. 3A). As homozygotes, most *osi*^Gal4^ individuals died throughout development (only 2/50 individuals emerged as adults, Fig. 3B), development was delayed although not arrested, confirming that reduced *osi* levels compromise survival. A proportion of *osi*^100B^/*osi*^Gal4^ individuals, on the other hand, reached adulthood.

### Reduced osi levels correlate with an arrest of larval development

Genome-wide RNA profiling revealed that *osi* transcript levels are normally moderate to high in every tissue starting at early development, becoming very high at around 12-h-old L3 instar larvae [27, 28]. The temporal correlation between the surge in *osi* expression in controls and the onset of lethality in the mutant (72h AEL), prompted us to assess *osi*’s relevance during development by an independent method. Thus, we resorted to the expression of *osi*^RNAi^, a RNAi line with no predicted OFF-target effects, in the context of *dicer 2* co-expression to increase RNAi efficacy. We first measured steady state levels of *osi* mRNA by qPCR in total RNA extracts from controls (*act*G4> +) and larvae expressing the RNAi driven by the *actin-*Gal4 driver (from now onwards referred to as *act*G4>*dcr2*;*osi*^RNAi^). Ubiquitous *osi* knockdown (*act*G4>*dcr2*;*osi*^RNAi^) resulted in over 80% reduction in overall *osi* mRNA levels (Fig. 3C).

Newly hatched larvae were placed in agar plates and were imaged every day to monitor progression of larval development for as long as experimental larvae were alive. The ubiquitous expression of *osi*^RNAi^ phenocopied the original mutant; *act*G4*> osi*^RNAi^ larvae displayed a similar feeding behavior and failed to grow and progress beyond the L1 stage. RNAi-mediated *osi* downregulation triggered complete lethality around 96h AEL (Fig. 3D).

### orsai is highly expressed in the larval fat body

**Fig. 4:**
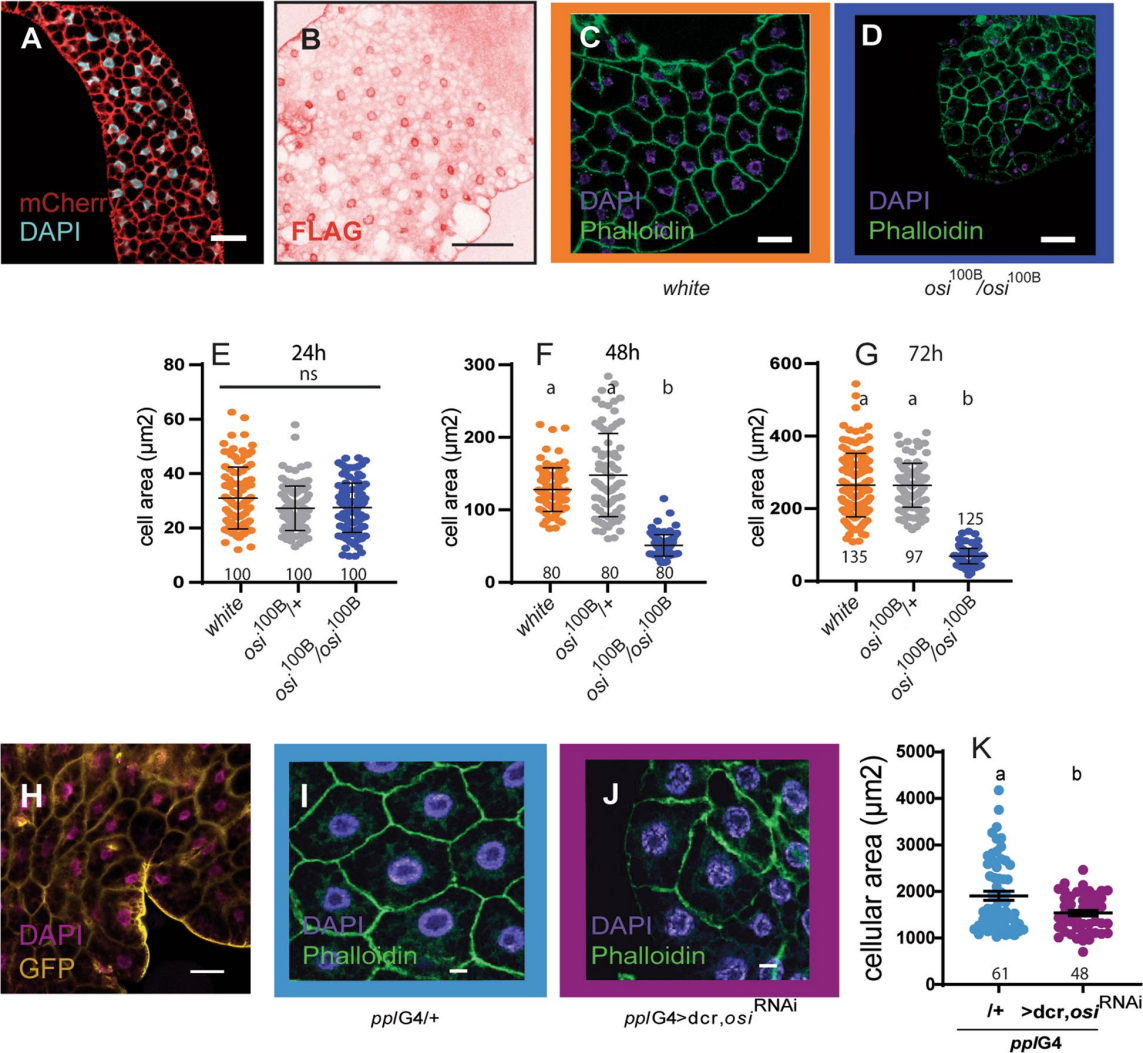
*osi* downregulation is associated with a smaller cell size. **A** Osi is expressed in the fat body. mCherry expression driven by *osi*^Gal4^ shows strong consistent signal in the fat body. **B** Confocal images of a fat body of larvae expressing Osi-FLAG under *act*G4, stained for Flag (in red). Scale bar represents 50 μm (**A**) and 100 μm (**B**). **C–G** *osi* mutants develop cellular defects in time. **C, D** Representative confocal images of fat body of control and mutant animals at 72h stained with DAPI (nucleus) and phalloidin (cell outline). Scale bar represents 20 μm. **E–G** Quantification of cellular area of larval fat bodies of 24h (**E**), 48h (**F**), and 72h (**G**) AEL individuals of control, heterozygous, and homozygous mutants. Five or six fat bodies were photographed per genotype on each time point. Dots in each graph represent individual cell sizes. **H** GFP directed by *ppl*G4 confirms fat body expression. Bars represent 20 μm. **I–K:** Phalloidin and DAPI stained fat bodies from 72h AEL control (**I**) and larvae expressing *osi*^RNAi^ under *ppl*G4 (**J**). Bars represent 10 μm. **K** Larvae expressing *osi*^RNAi^ show reduced cell size. Quantitation of cell size of fat body cells from 72h AEL control and larvae expressing *osi*^RNAi^ under *ppl*G4. Three fat bodies were photographed per genotype. Dots in each graph represent individual cell sizes. All graphs describe the mean ± SEM. In **E**, **F**, and **G**, a Kruskal-Wallis with Dunn’s multiple comparisons test was performed. In **K**, a Mann-Whitney test was performed. All panels: the total number of observations is indicated under each data set. A Shapiro-Wilk test was used for normality assessment (see Additional file 2). Different letters indicate significant differences, *p*<0.05; treatments sharing any letter are not statistically different. The total number of samples analyzed is detailed below the corresponding dataset. All datasets and statistical analysis on which the conclusions are based are included in Additional file 2

Publicly available data predicts that *osi* is expressed in most tissues. Within the larvae, levels are particularly elevated in the fat body (up to three times, according to FlyAtlas [27]), To independently confirm *osi* expression in the larval fat body, we resorted to *osi*^Gal4^ to drive expression of a membrane associated mCherry. As shown in Fig. 4A, *osi*^Gal4^ is expressed throughout the fat body. Interestingly, expression of a tagged Osi version suggests this protein localizes in the proximity of the nucleus in control fat body cells (Fig. 4B).

### Partial loss of osi function reduces cell size

The observation that Osi was localized in fat body cells prompted us to examine the consequences of *osi* loss-of-function in this tissue. To test the possibility that depleted Osi could be associated with reduced cellular size, we dissected the fat body of controls, heterozygous and homozygous mutants at 24, 48, and 72h AEL. Figure 4C–G shows that while homozygous mutant cells were similar in size to matched controls at 24h AEL, their size was significantly reduced at 48h. These differences were even more pronounced at 72h AEL.

We further explored this possibility through expression of *osi*^RNAi^ in fat body cells by means of the *ppl*G4 driver, whose expression at this stage was first confirmed (Fig. 4H). Not surprisingly given the phenotypes observed in the mutants, Osi downregulation triggered a reduced cellular size (Fig. 4I–K). These results suggest that impaired *osi* function leads to a defective control of cell and/or organ size.

### Mosaic analysis uncovers a cell autonomous role for Osi

**Fig. 5:**
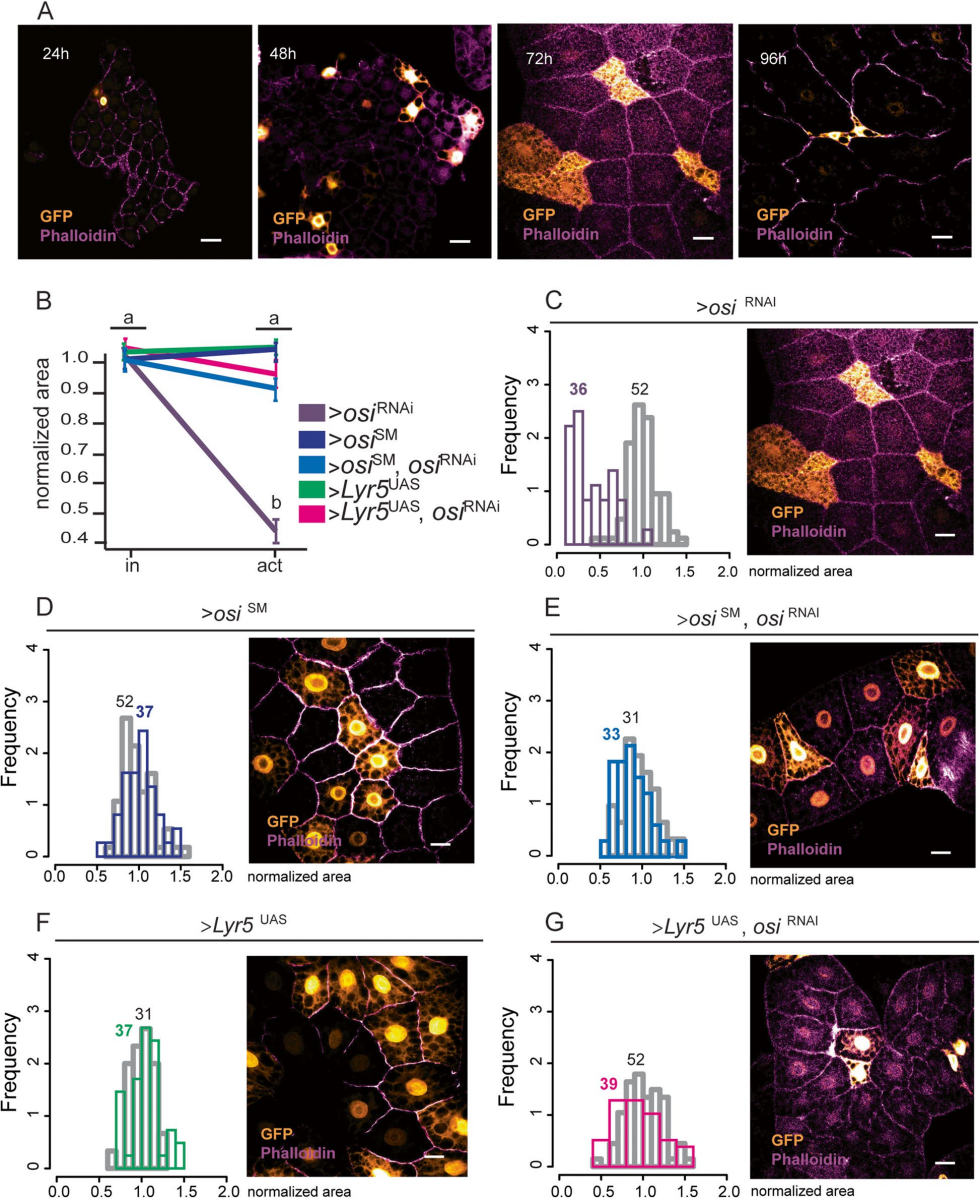
ETFRF1/LYRm5 rescues cellular and viability defects associated with *osi* knockdown. **A** Confocal microscopy images showing the progression of cell growth phenotypes in fat body cells expressing *osi*^RNAi^ (GFP+, golden cells) that are growing among control fat body cells (GFP−). Images were taken every 24h until pupation. **B** Cell size defects are fully rescued upon expression of ETFRF1/LYRm5 or Osi^SM^. The graph shows the normalized area for GFP+ (active)/GFP− (inactive) fat body cells. Kruskal-Wallis test with Dunn’s multiple comparisons test was performed. Three fat bodies per genotype were analyzed, for each sample, data was normalized to the mean of the control (inactive cells) of the corresponding genotype, in order to independently compare the absolute value. Shapiro-Wilk test was used for normality assessment (see Additional file 2). Kruskal-Wallis with Dunn’s multiple comparisons test was used to determine significance. **C–G** Images were taken at 72h AEL. Left panels: Frequency distribution of fat body cells in which the flipase was inactive (grey bars) or active (colored bars) and thus activating expression of *osi*^RNAi^ (**C**, the figure shown in panel **A** is included for direct comparison), *osi*^SM^ (**D**), *osi*^RNAi^, *osi*^SM^ (**E**), LYRm5 (**F**), or LYRm5, *osi*^RNAi^ (**G**). The number of cells analyzed per genotype is included in the graph. Right panels: Representative images of fat body cells from individuals of the indicated genotypes. All photographs include phalloidin-rhodamine staining (purple) to mark cell outlines. Cells expressing the constructs of interest are labeled in gold. Bars represent 20 μm. The total number of samples analyzed is mentioned below the corresponding panel. All datasets and statistical analysis on which the conclusions are based are included in Additional file 2

Depletion of Osi arrests larval development. To dissect Osi’s role in the context of a viable organism, we generated mosaic animals in which somatic cell clones expressed *osi*^RNAi^ along with a GFP reporter using the site-specific recombination flp/FRT system [29]. This method generates cell clones expressing Gal4 (and thus, GFP along with *osi*^RNA*i*^) once a heat shock triggers recombination between cis-acting sites. We applied this method to obtain clones in the fat body (Fig. 5). Twenty-four-hour AEL larvae were exposed to a very brief heat shock, and their fat body was dissected at 24-h intervals. The frequency of clones is dependent on the duration of the heat shock and larval age. Figure 5A illustrates the results obtained. Initially, no clear differences in cell size or morphology could be observed between control (GFP−) and *osi*^RNAi^ (GFP+) expressing cells (Fig. 5A, 24 and 48h). However, 72h after the heat pulse, GFP+ cells were smaller in size and also displayed an abnormal morphology, which became more dramatic later on (96h), when GFP+ clones were barely detectable, prior to their complete disappearance. Thus, mosaic analysis confirmed prior observations suggesting that Osi is relevant for the control of cell size and revealed that Osi acts in a cell-autonomous manner.

### Human LYRM5/ETF regulatory factor 1 (ETFRF1) rescues Osi loss of function

As shown in Fig. 2, Osi shares 50% identity with human ETFRF1/LYRm5. In vitro, ETFRF1/LYRm5 was found to inhibit ETF by promoting the removal of flavin from the ETF holoenzyme, thus potentially regulating the rate of β-oxidation [25]. To investigate whether the human protein could counteract the cell-autonomous phenotypes associated with loss of Osi function, codon-optimized ETFRF1/LYRm5 was expressed in the context of *osi*^RNAi^ in the same experimental setting already described (Fig. 5A). Figure 5B–G shows that, while clonal expression of *osi*^RNA*i*^ results in a statistically significant reduction of cell size, co-expression of human ETFRF1/LYRm5 rescues this defect to a large extent, indistinguishable from the one achieved through the expression of a RNAi-resistant *osi* (*osi*^SM^), supporting the notion that ETFRF1/LYRm5 is the human ortholog of Osi.

**Fig. 6:**
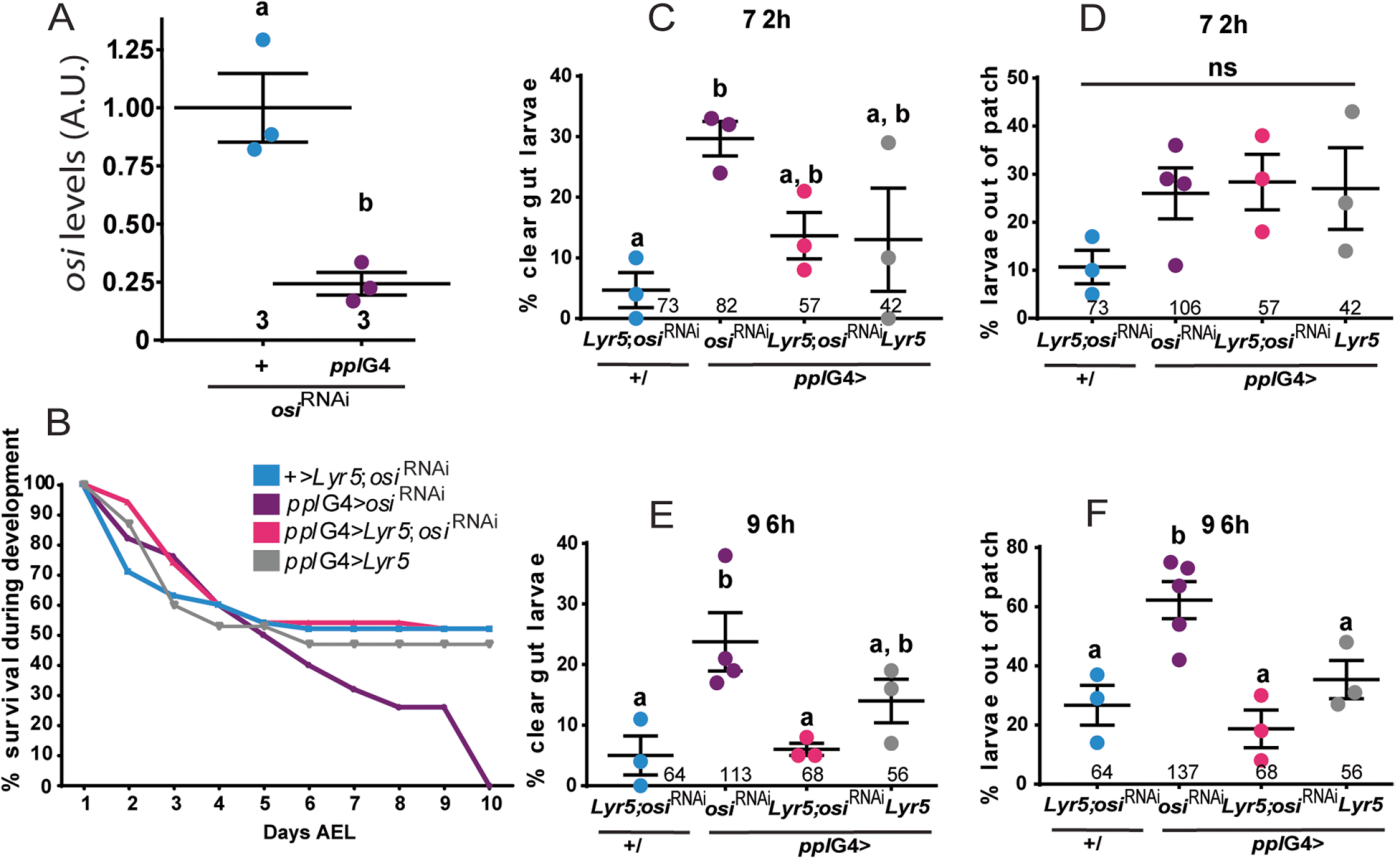
Expression of human ETFRF1/LYRm5 rescues lethality associated with *osi* downregulation in the fat body. **A** qPCR analysis on RNA extracted from whole larvae shows efficient reduction of *osi* levels when *osi*^RNAi^ is expressed under *ppl*G4 at 96h AEL. The total number of replicas/genotype is included under each data set; each dataset consisted of 30 larvae pooled together. A two-tailed *T*-test was used to analyze statistical significance. **B** Survival curve for *ppl*G4> *osi*^RNAi^*, ppl*G4> LYRm5;*osi*^RNAi^*, ppl*G4> LYRm5 and *+/* LYRm5;*osi*^RNAi^ genotypes*.* Animals were surveyed up to 10 days AEL, when all living individuals had arrived to the pupal stage. Survival indicates the percentage of living individuals. The experiment was repeated twice, a representative experiment is shown. **C, E** Percentage of the population observed outside of the yeast patch. **D, F** Percentage of clear gut larvae. Larvae were monitored at 72h (**C, D**) and 96h AEL (**E**, **F**). Each dot represents the percentage per experiment. The total number of animals is indicated. One-way ANOVA with Bonferroni’s multiple comparisons was performed. All panels: Shapiro-Wilk test was used for normality assessment (see Additional file 2). Different letters indicate significant differences, *p*<0.05; treatments sharing any letter are not statistically different. All graphs describe the mean ± SEM. The total number of analyzed samples is included below the corresponding panel. All datasets and statistical analysis on which the conclusions are based are included in Additional file 2

**Fig. 7:**
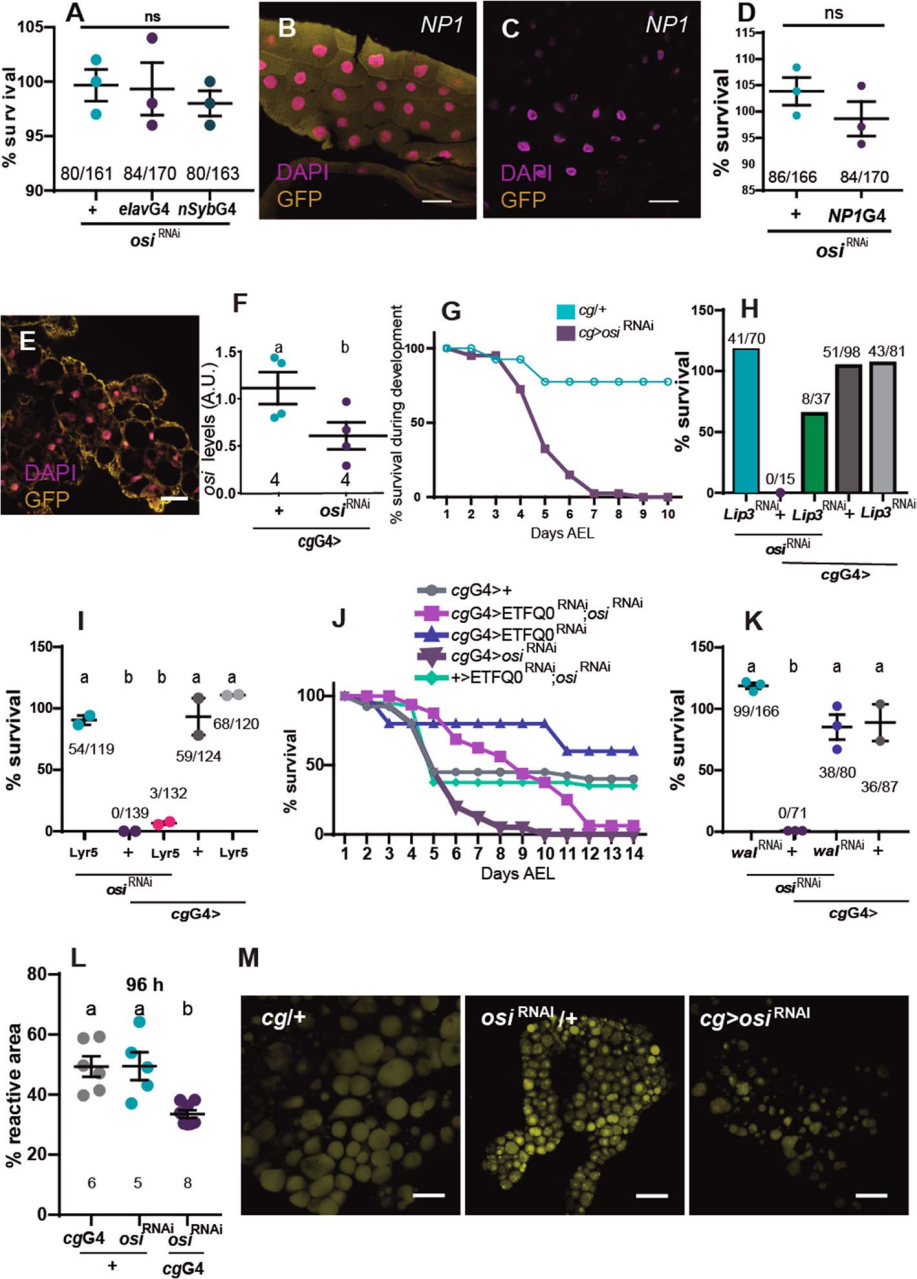
A key role for *osi* in the fat body. **A** *osi* downregulation in the nervous system using *elav*G4 or *nSyb*G4 does not affect viability. *N*=3. **B–D** *osi* downregulation in the gut does not affect viability. NP1G4 is expressed in the gut of 72h AEL larvae. **B, C** show GFP driven by NP1G4 confirming its expression in the gut (**B**) but not in the fat body (**C**). *N*=3. **E–M** *cg*Gal4 recapitulates *osi*-related phenotypes. **E** *cg*Gal4 directed expression of GFP to the fat body. **F** qPCR shows *osi* levels are reduced about 50%. *N*=4; each replica included 20 larvae. **G** Survival indicates the percentage of living individuals. *N*=2, one representative experiment is shown. **H** *Lip*3 downregulation in the fat body accounts for partial survival. Adult eclosion of the indicated genotypes was quantified. The graph shows the proportion of animals obtained relative to the expected (“Methods”). *N*=1. **I**: LYRm5 expression in the fat body partially rescues the lethality associated to reduced *osi* levels. Adult eclosion was quantified. *N*=2. **J** Downregulation of *osi* and EtfQ0 in the *cg*G4 pattern progress through development, molt and pupate; even though no viable adult eclosed. A representative experiment (*N*=2) is included. **K** *wal* (EtfA) downregulation in the fat body fully rescues survival with reduced *osi* levels. *N*=3. **L, M** *osi* downregulation in the fat affects lipid droplet content. **L** Reactive area of lipid droplets stained with BODIPY was quantified. Six to eight individuals were analyzed per genotype. Each dot represents the mean reactive area per animal. **M** representative images. All panels: different letters indicate significant differences, *p*<0.05; treatments sharing any letter are not statistically different. The total number of animals analyzed is indicated in **D**, **H**, **I**, and **K**. A Shapiro-Wilk test was used for normality assessment. A *T*-test or one-way ANOVA with Bonferroni’s multiple comparisons was performed (see Additional file 2). The total number of samples analyzed is described below each dataset. All datasets and statistical analysis on which the conclusions are based are included in Additional file 2

As already mentioned, larvae with ubiquitous *osi*^RNAi^ expression die at first instar, judged by the morphology of the mouth hooks. To investigate the impact of *osi* knockdown restricted to the fat body (*ppl*G4), we examined their progression through developmental stages; interestingly, about 25% of *osi-*depleted animals reached pupal stages, although no adult ever emerged (Figs. 6A and 7I), underscoring an essential function in the organ that is considered the fly equivalent to the vertebrate adipose tissue and liver. Moreover, ETFRF1/LYRm5 expression almost completely rescued the lethality triggered by *osi*^RNAi^ in the context of *ppl*G4 (from 0% survival rate in *ppl*G4>*osi*^RNAi^ up to 67% in *ppl*G4>LYRm5; *osi*^RNAi^), reinforcing the possibility that ETFRF1 and Osi play a similar biochemical function. Taking into account the behavioral phenotype of the insertional *osi* mutants, we entertained the possibility that lack of Osi function in the brain could account for this abnormal behavior. Pan-neuronal *osi*^RNAi^ expression was then examined (through *elav*G4 and *nSyb*G4 drivers). However, no developmental defects or lethality was observed under these conditions, supporting a non-neuronal trigger for this change in food-seeking behavior (Fig. 7A).

To further examine the function of *osi* within the fat body, we closely monitored feeding behavior upon *osi* knockdown restricted to this organ. qPCR analysis confirmed the effectivity of *osi* downregulation restricted to the fat body (Fig. 6A). Osi-depleted animals exhibited a survival rate similar to the controls up to 72h AEL; however, after 96h AEL, lethality became pronounced in *osi*^RNAi^ individuals soon after control animals started to pupate (Fig. 6B). This narrow window was selected to further analyze food-seeking behavior. Figure 6C–F shows that *osi*^RNAi^ animals phenocopy *osi*^100B/100B^ mutant behavior both at 72h and 96h AEL, namely, a high percentage displayed a clear gut and was found outside of the nutritious yeast patch compared to controls. These results reinforce the notion that aberrant behavior is triggered by depleted Osi function outside of the brain, opening the possibility that Osi loss of function in the fly adipose tissue/liver alters basal metabolism which, in turn, leads to the dramatic behavioral change. Such possibility is further supported by the observation that LYRm5 expression not only rescues abnormal feeding patterns but also gives rise to an increased survival rate.

Given that *ppl*G4 is expressed in additional tissues (of particular interest is the report of expression in the gut, [30]), we examined whether NP1G4 (a commonly used gut driver with undetectable expression in the L3 fat body, Fig. 7B,C) phenocopied *ppl*G4 directed *osi* knockdown; however, survival was not compromised under these conditions (Fig. 7D). Next, we repeated the functional analysis with an additional fat body-specific driver (*cg*Gal4, Fig. 7E). Consistent with a key role for *osi* in the fat body as opposed to the gut, *cg*G4>*osi*^RNAi^ triggered early lethality (Fig. 7G). Taken together, these data demonstrate that the aberrant behavior associated with Osi depletion is a consequence of the alteration of a metabolic program within the fat body that only in time affects food-seeking behavior.

### osi mutants have impaired cellular metabolism

**Fig. 8:**
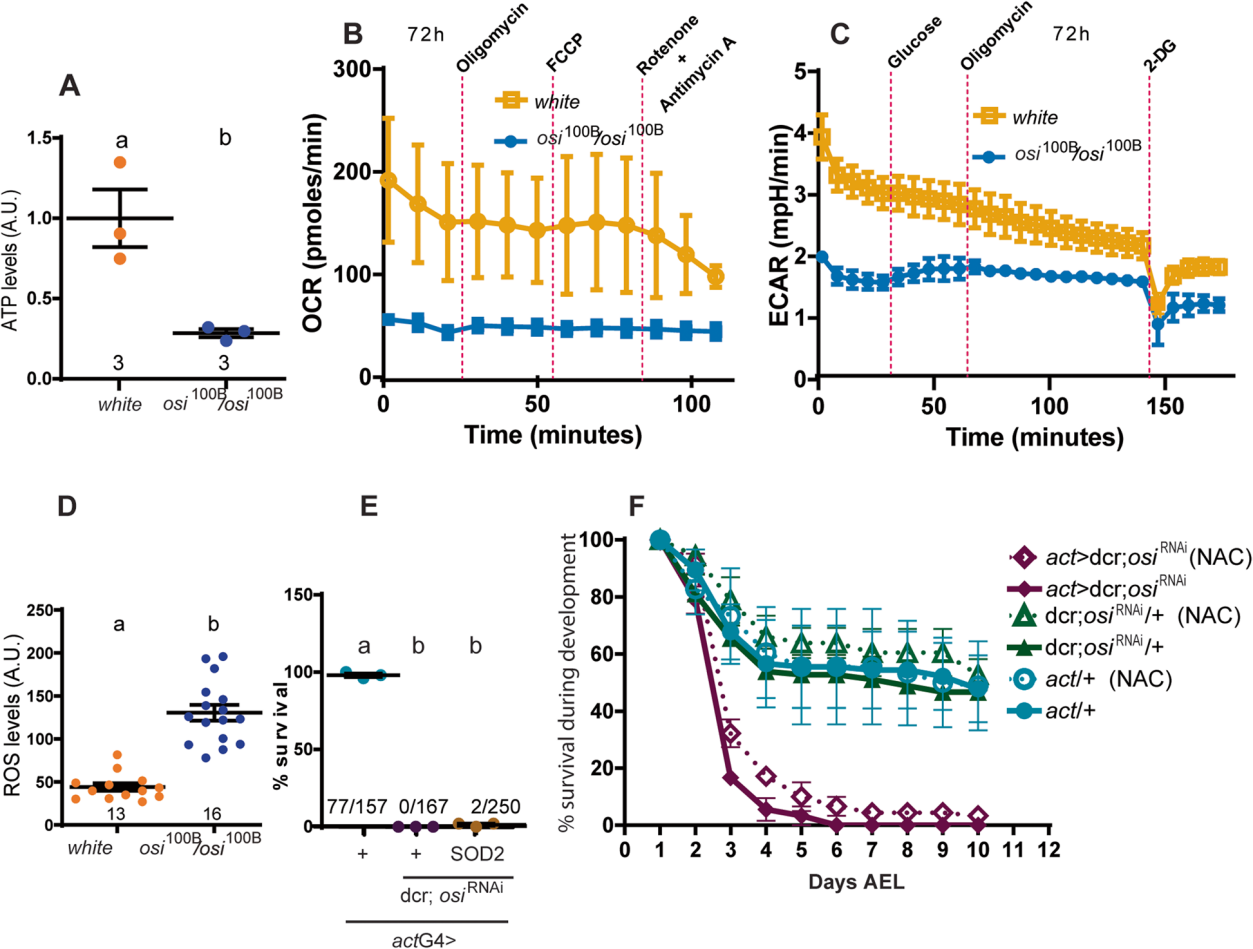
*osi* loss of function produces a metabolic defect not rescued by antioxidants. **A** Mitochondrial ATP levels normalized to total protein. Three independent samples were evaluated. Two-tailed *t*-test was performed. **B** Mitostress analysis shows a reduction in oxygen consumption (OCR) in *osi* mutants at 72h AEL. Data was normalized to the number of larvae. Two independent samples were evaluated. **C** Glycostress analysis reveals a reduced glycolytic metabolism in *osi* mutants as well as an altered response to glucose intake at 72h AEL. Data was normalized to the number of larvae. Six independent samples were examined. **D** Dihydroethidium (DHE) was employed to detect ROS by fluorescence microscopy in 24h AEL larvae. Animals from 2 different plates were used. The total number of fat bodies analyzed are indicated. A two-tailed *t*-test was performed. **E** Sod2 expression in *osi-*depleted animals using *act*G4. Three independent samples were taken into account. A one-way ANOVA with Bonferroni’s multiple comparisons was performed. The total number of animals per condition is indicated. **F** Addition of the antioxidant NAC to the food did not improve survival of *osi*-depleted animals. The experiment was repeated twice. All panels: different letters indicate significant differences, *p*<0.05; treatments displaying the same letter (alone or in combination) are not statistically different. A Shapiro-Wilk test was used for normality assessment (see Additional file 2). The total number of samples analyzed is described below the corresponding panel. All datasets and statistical analysis on which the conclusions are based are included in Additional file 2

Reduced *osi* levels elicited developmental arrest at a stage of active growth, coincident with times of increased energy demand. This observation coupled with the fact that *osi* dysfunction leads to a reduced cell size in a tissue that has key metabolic functions, prompted us to consider that lethality could be linked to a defective cellular metabolism. Mitochondrial extracts were prepared from 72h AEL homozygote *osi*^100B^ larvae along with controls. Fresh extracts were then assayed for protein content and ATP production employing a quantitative bioluminescence kit. As shown in Fig. 8A, mutant extracts generated ATP levels three times lower than those of the controls, confirming that reduced Osi correlates with an impaired ATP production. We next estimated oxygen consumption rate (OCR) through a ¨Mitostress¨ protocol in control and mutant larvae, which relies on the sequential addition of inhibitors of the respiratory chain to assess the function of specific complexes. To improve access of the inhibitors, an open larval preparation where all the organs are exposed was selected. However, no changes in OCR became detectable in control preparations, with the exception of the addition of Rotenone/Antimycin A that resulted in the expected reduction in OCR, in line with previous reports describing similar body wall preparations [31]. Although these results should be taken cautiously, OCR was reduced in homozygous *osi*^100B^ mutants (Fig. 8B), indicative of a reduced respiratory capacity. Next, we measured the extracellular acidification rate (ECAR) under a “Glycostress” assay; these experiments showed an overall defective glycolytic capacity in *osi*^100B^ mutants (Fig. 8C).

This impaired metabolic function is often associated with the accumulation of charged cytotoxic species that affect membrane stability and could induce the formation of superoxide anions, thus contributing to the generation of cellular reactive oxygen species (ROS) [32]. To investigate this possibility, a chemical probe called dihydroethidium (DHE) was employed to detect superoxide radicals by fluorescence microscopy in intact larval tissue [33, 34]. Interestingly, homozygous *osi*^100B^ larvae showed a 2-fold increase in overall superoxide radical levels as reported by this probe (Fig. 8D); such increase in ROS levels could per se exceed the capacity of intrinsic antioxidant systems and therefore lead to oxidative stress and cell damage. To investigate this possibility, we resorted to the expression of superoxide dismutase (SOD). SOD catalyzes the conversion of superoxide anion radicals to hydrogen peroxide which is in turn converted to molecular oxygen and water by catalase. Overexpression of SOD and catalases retards accumulation of oxidative damage associated with aging [35, 36], as well as prevents some of the deleterious effects in fly models of disease [37]. To explore the possibility that increased superoxide levels could contribute to the characteristic lethality associated with depleted Osi, we expressed *sod2* in the context of the ubiquitous expression of *osi*^RNA*i*^ (*act*G4> *osi*^RNAi^*, Sod2*). Only a marginal rescue of lethality was observed; very few individuals progressed through development into adult stages, albeit not in the expected proportion, suggesting that increased superoxide levels have a minor contribution to *osi*’s phenotype (Fig. 8E). To further explore the relevance of free radicals to the mutant phenotype, animals were grown in N-acetylcysteine supplemented food (NAC, a potent antioxidant); under these conditions, the survival rate was similar to the one observed upon Sod2 overexpression (Fig. 8F).

Overall, this data suggests that depleted Osi function unbalances energy metabolism.

### Deregulated lipid catabolism underlies cell-autonomous and systemic phenotypes

**Fig. 9:**
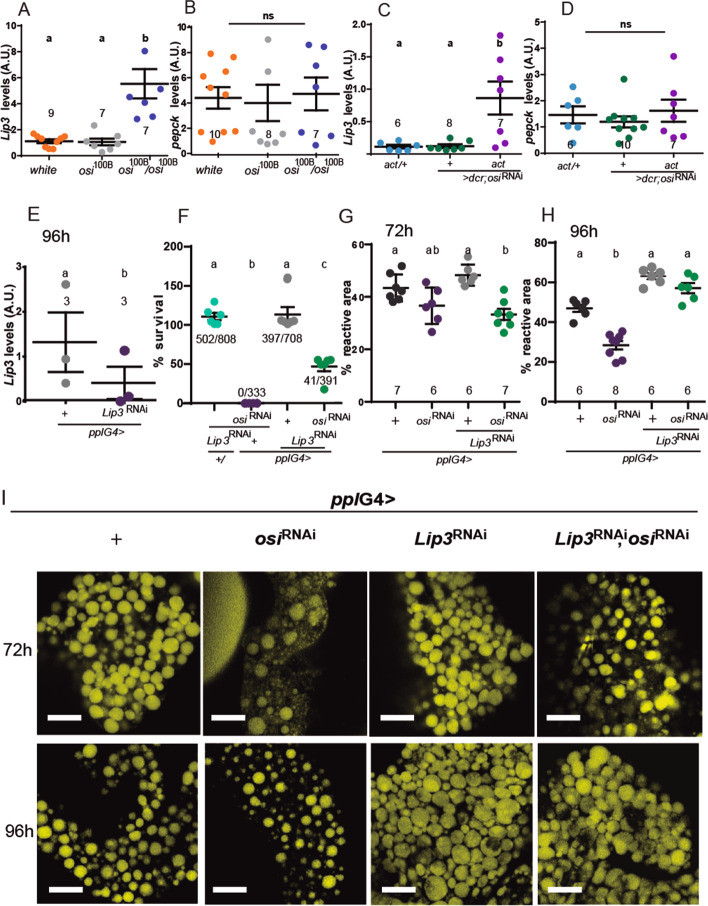
Preventing excessive fat body lipid catabolism rescues the behavioral effect triggered by *osi* downregulation. **A–D** Quantitation of *Lip3* and *pepck* mRNA levels in mutants (**A, B**) or larvae with ubiquitous RNAi expression (**C, D**). Data shows a clear increase in *Lip3* with no effect on *pepck* levels. The total number of replicas/genotype is included; each dataset consisted of 20 larvae pooled together. A Shapiro-Wilk test was used for normality assessment; if normality was confirmed, a one-way ANOVA with Bonferroni’s multiple comparisons test was performed. For nonparametric assessment, Kruskal-Wallis with Dunn’s multiple comparisons test were performed (see Additional file 2). **E** qPCR analysis on RNA extracted from whole larvae shows efficient reduction of *Lip*3 levels when *Lip3*^RNAi^ is expressed under *ppl*G4 at 96h AEL. The total number of replicas/genotype is included; each dataset consisted of 20 larvae pooled together. A two-tailed *t*-test was performed. **F** *Lip3* downregulation partially rescues *osi*^RNAi^ lethality. Six independent experiments were performed; the total number of animals assessed are indicated. **G–I** Confocal images showing fat body cells stained with BODIPY (golden lipid droplets) for control, *osi*^RNAi^, *Lip3*^RNAi^, and *Lip3*^RNAi^, *osi*^RNAi^ expressing animals under *ppl*G4 from 72 and 96h AEL. Bars represent 20 μm (**I**). The percentage of area covered by lipid droplets or “reactive area” is quantitated in **G** for 72h and **H** for 96h AEL. Each dot represents the mean area of each fat body analyzed. The total number of fat bodies per condition is indicated. All panels: Shapiro-Wilk test was used for normality assessment. Graphs display mean ± SEM. Different letters indicate significant differences, *p*<0.05; treatments sharing any letter are not statistically different. The total number of samples analyzed is described below the corresponding panel. All datasets and statistical analysis on which the conclusions are drawn are included in Additional file 2

To gain more insight into the consequences of reduced Osi, we measured *Lipase 3* (*Lip3*) mRNA levels, a key enzyme in lipid catabolism, which is increased upon starvation [17, 38]. In effect, *Lip3* levels were upregulated in *osi*^100B/100B^ mutants as well as in *act*G4>*osi*^RNAi^, while *Pepck1* levels, encoding an enzyme that regulates carbohydrate catabolism [39], were not modified at 72h AEL (Fig. 9A–D). We then tested the relevance of *Lip3* upregulation to *osi-* related phenotypes through tissue-specific RNAi downregulation (Figs. 7H and 9F–H). Noteworthy, *Lip3*^RNAi^ expression in the context of *osi*^RNAi^ partially rescued lethality, suggesting that an increased lipid catabolism is clearly linked to the developmental arrest (Fig. 9F). If this was the case, then impaired *osi* function could correlate with a reduction of lipid reserves. To shed light on this possibility, we stained fat bodies with BODIPY, a fluorescent dye for the assessment of cellular neutral lipid content [40]. As predicted, lipid content was dramatically decreased in tissue-specific knockdowns at 72 and 96h AEL (*ppl*G4>*osi*^RNAi^, Fig. 9G–I and S2L-M). We reasoned that if reduced Osi function increases the rate of lipid catabolism through excessive Lip3 activity, *Lip3* knockdown in the context of reduced Osi levels could ameliorate the lipid storage phenotype. Interestingly, Fig. 9 shows that to be the case, whereby concomitant downregulation of the two proteins rescued lipid droplet (LD) content to background levels (Fig. 9G–I). Although we cannot rule out the contribution of fasting to increased *Lip3* levels, we favor the interpretation of a more direct link since reducing *Lip3* levels in the context of *osi* knockdown associates with increased survival which would not be the expected outcome when impairing a fasting response in the context of reduced feeding.

**Fig. 10:**
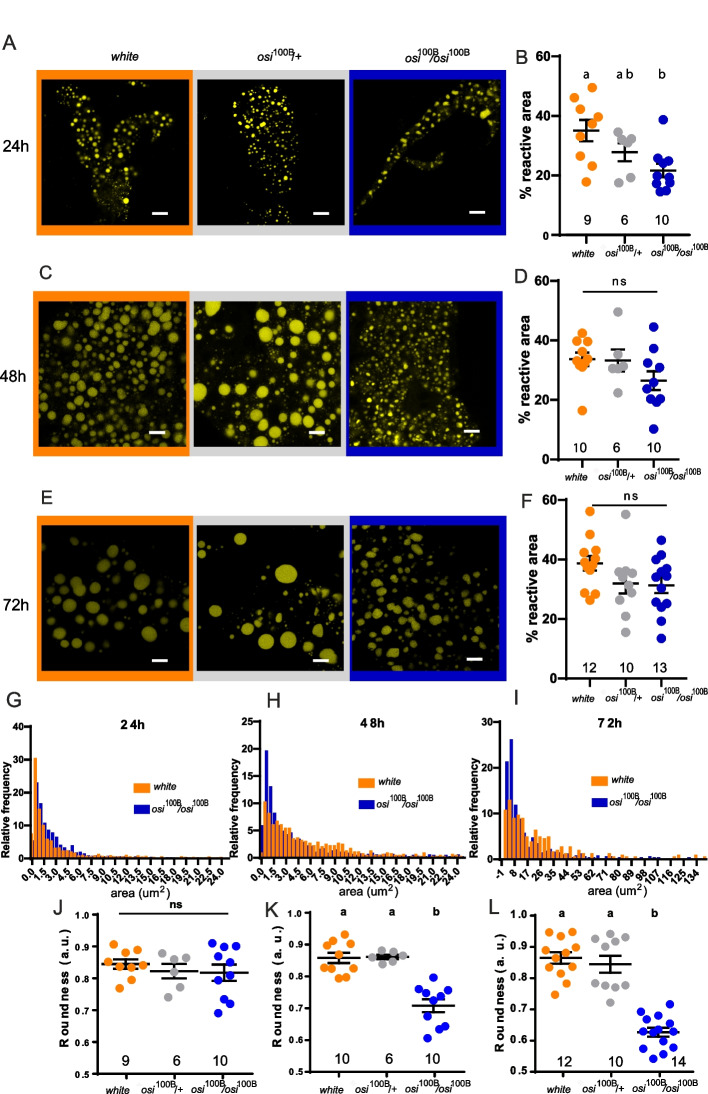
*osi* mutants have a progresive shift in LD size. **A, C, E** Downregulation of *osi* changes the distribution of lipid droplet size. The total area covered by lipid droplets (reactive area) is not significantly different among genotypes; in the mutant, there is a shift towards smaller lipid droplets. Confocal microscopy images showing lipid staining in fat body cells of controls (+/+), *osi*^+/−^ (+/−) and *osi*^100B/100B^ (^100B/100B^) stained with BODIPY (golden lipid droplets) for 24h (**A**), 48h (**B**), and 72h (**C**) AEL. **B, D, F** Percentage of area covered by fluorescence or “reactive area” for lipid droplets of animals of 24 (**B**), 48 (**D**), and 72 (**F**) hours AEL. Each dot represents the mean area of each fat body analyzed. The total number of fat bodies per condition is indicated. **G, H, I** Frequency distribution of lipid droplet size from animals of 24 (**G**), 48 (**H**), and 72 (**I**) hours AEL. Only controls (+/+) and *osi*^100B/100B^ are shown to facilitate direct comparison. **J, K, L** Lipid droplet roundness measured by ImageJ as a way of assess structural integrity for 24h (**J**), 48h (**K**), and 72h (**L**) AEL. All panels: Shapiro-Wilk test was used for normality assessment; when normality was confirmed, a one-way ANOVA with Bonferroni’s multiple comparisons was performed. For nonparametric assessment, Kruskal-Wallis with Dunn’s multiple comparisons test were performed (see Additional file 2). Different letters indicate significant differences, *p*<0.05; treatments sharing any letter are not statistically different. Scale bars represent 10 μm. The total number of samples analyzed is mentioned below in the corresponding column. All datasets and statistical analysis on which the conclusions are based are included in Additional file 2

Additionally, we tested if lipid content was decreased in *osi*^100B/100B^ mutants (Fig. 10). Despite no significant reduction in total area was detectable (Fig. 10B, D, F), we did find a clear change in the individual size of LDs. As shown in Fig. 10G–I, the distribution of lipid droplet size shifted towards a higher proportion of smaller ones that were increasingly represented over time in the mutants. In addition, at the end of the experiment, mutant lipid droplets exhibited an aberrant morphology of these otherwise round shaped organelles, underscoring an increased use of lipid reserves (Fig. 10J–L). The apparent subtler phenotype in *osi*^100B/100B^ mutants might simply reflect that those observations were performed at an earlier timepoint during larval development. Nevertheless, considering that *Lip3* downregulation per se partially compensates for loss of Osi function (Fig. 9G–I), we propose that the overuse of lipid reserves is a key component of the phenotype observed.

**Fig. 11:**
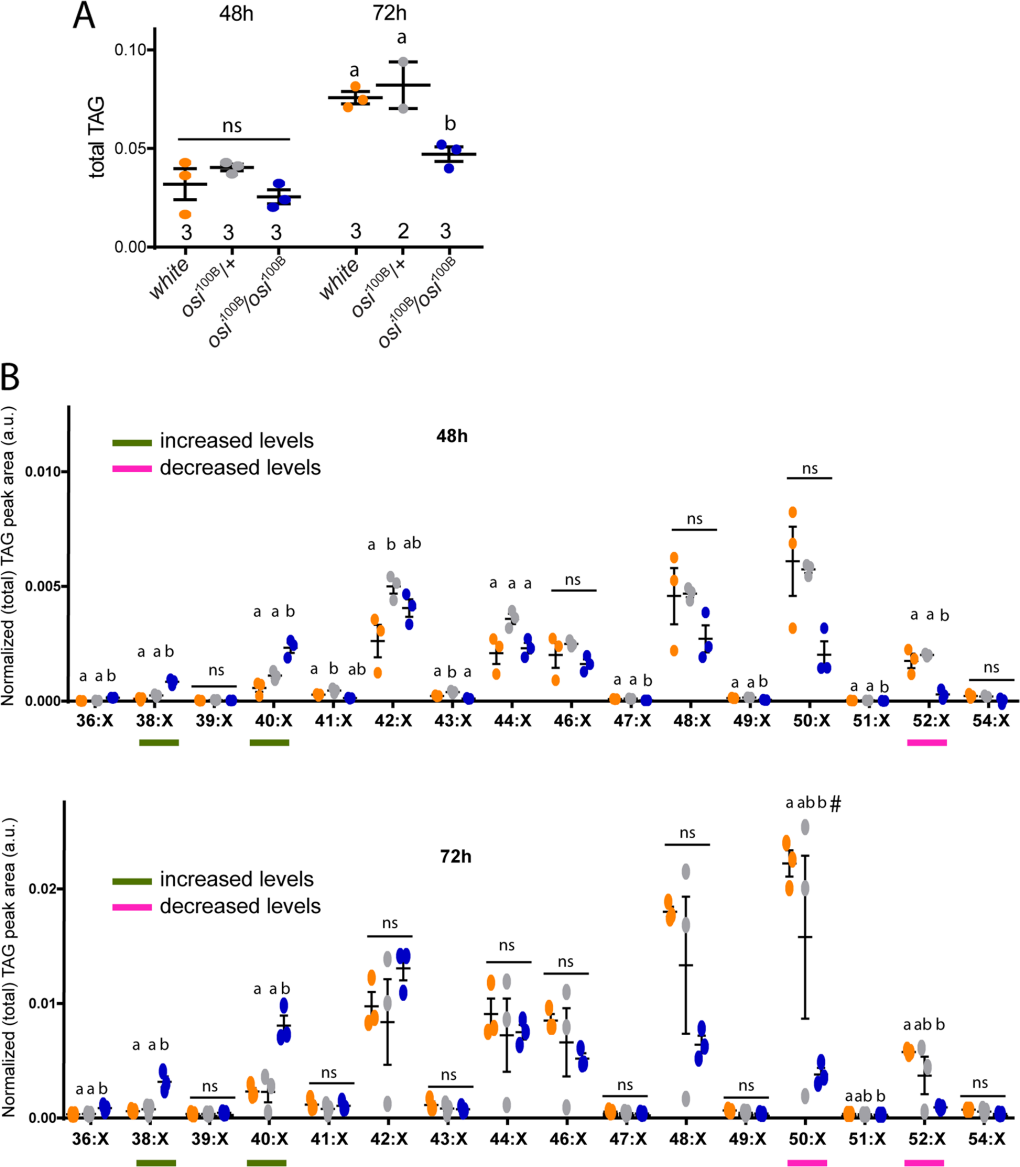
Reduced *osi* levels alters the storage lipid content and composition. **A** Assessment of total TAG content shows no significant differences at 48h but a clear decrease at 72h in mutant animals compared to controls. **B** For clarity purposes, TAGs with same carbon number but different degree of saturation were grouped together. TAG groups are differentially affected. The differential profile of TAG groups at 48 and 72h show that *osi*^100B^ mutants have increased low molecular weight TAG groups (additive carbon number 38 and 40), and a corresponding decrease in high molecular weight TAG groups (additive carbon number 50, 52, trend also 48). Three independent samples were analyzed. Each sample included 50 larvae pooled together. All panels: different letters indicate significant differences, *p*<0.05; treatments sharing any letter are not statistically different. A Shapiro-Wilk test was used for normality assessment; if normality was confirmed, a one-way ANOVA with Bonferroni’s multiple comparisons was performed. For nonparametric assessment, Kruskal-Wallis with Dunn’s multiple comparisons test were performed (see Additional file 2). The “#” symbol in last panel for 50:X group represents a strong tendency (*p*= 0.0523). The total number of samples analyzed is included below the corresponding panel. All datasets and statistical analysis on which the conclusions are based are included in Additional file 2

The striking change in LD size and shape when Osi function is compromised prompted us to examine lipid content in more detail. Among neutral storage lipids, triacylglycerol (TAGs) are the major constituents of LDs and most abundant fatty acid source for energy production as well as signaling lipid biosynthesis [41]. We performed mass spectrometry-based lipidomics in control and *osi* mutant animals at 48 and 72h AEL. The total TAG content of *osi*^100B^ larvae at 72h AEL was significantly reduced compared to controls, but this tendency was already present at the earlier timepoint (Fig. 11A). Interestingly, this decrease was not the result of a general reduction in every species; in fact, a clear reduction was observed in the TAG species composed of common long-chain fatty acids (LCFAs), particularly with total carbon number C52, C50, and C48, while lower molecular weight TAG species composed of shorter LCFAs and medium chain FAs (total carbon numbers C40, C38, and C36) were significantly increased. This differential change of the TAG profile was already evident at 48h AEL and become more pronounced at 72h AEL (Fig. 11B). Currently, we cannot judge whether these differences arise from *osi*-dependent developmental changes of TAG biosynthesis or breakdown. However, given that at this time in development, TAGs are not commonly used as the source of energy but as building blocks for membrane production and maintenance as well as a source of signaling molecules, this finding opens the possibility that changes in particular TAG groups may contribute to the complex array of phenotypes associated with reduced Osi levels [12].

### Epistasis analysis reveals a role for Osi at the center of lipid metabolism

ETFRF1/LYRm5 has been shown to negatively regulate the electron transfer flavoprotein complex (ETF) in vitro [25], suggesting that Osi provides a modulatory step in the response to the metabolic state of the cell. The ETF complex (including Walrus/EtfA and a yet to be defined EtfB subunit) is in charge of shuttling the electrons generated during mitochondrial fatty acid and amino acid catabolism by means of the electron transfer flavoprotein ubiquinone oxidoreductase (EtfQO) [42].

**Fig. 12:**
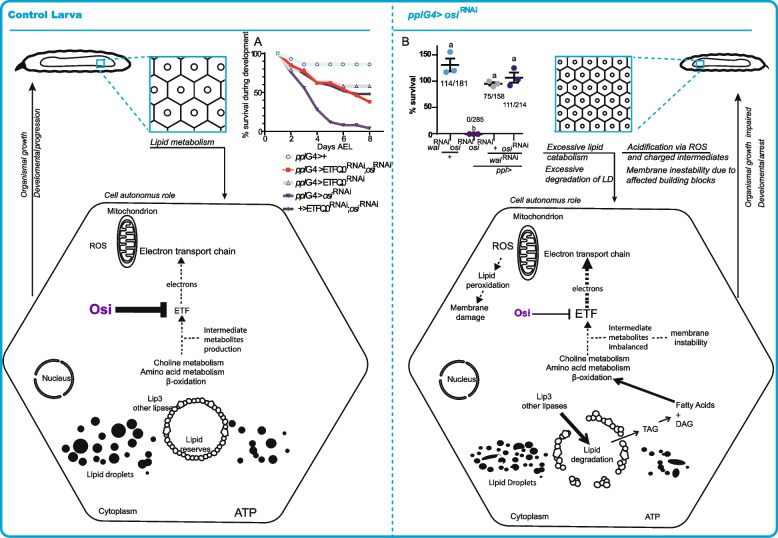
A model for Osi function. Comparison between fat body metabolism in a control and *osi*-depleted background. Depleted Osi/ETFRF1 levels are associated with activation of Etf/EtfQO, increasing electron flow to CoQ, with the consequent retrograde electron transfer, leading to an increase in ROS levels and unbalanced intermediate metabolites, which potentially alter the integrity and physical properties of all membranes. These changes in lipid droplet membrane would provide access to lipases, increasing lipid degradation and depleting general energy reserves, represented as a reduced lipid droplet size in the right panel. Eventually, this reduced lipid storage leads to a cell-autonomous decrease in cell size, altering the normal development and triggering a change from continuous feeding to an active food-seeking behavior even in the presence of nutritious sources. Thus, Osi links lipid catabolism to cell growth signaling essential during early development. **A** Downregulation of EtfQ0 rescues *osi*-related developmental phenotypes, albeit the animals do not reach adulthood. The experiment was repeated three times. A representative one is shown. **B** Downregulation of *walrus*, *Drosophila’s* equivalent to Etf subunit, rescues *osi*-related fat body phenotypes to a large degree. Animals become viable adults. Dots represent the percentage of survival. The total number of animals assessed is indicated. A one-way ANOVA with Bonferroni’s multiple comparisons was performed. Three independent experiments were performed. Different letters indicate significant differences, *p*<0.05; treatments sharing any letter are not statistically different. The total number of samples analyzed is listed below the corresponding panel. All datasets and statistical analysis on which the conclusions are based are included in Additional file 2

To inquiry whether arrested development could derive from a deranged β-oxidation, we explored if reducing Etf-Q0 or ETFA could restore such balance. Thus, we combined expression of Etf-Q0^RNAi^ and *osi*^RNAi^ and monitored progression throughout development. Despite no viable adults emerged, reducing Etf-Q0 levels in the context of *osi* knockdown rescued the developmental arrest up to puparium formation (Figs. 7J and 12A). We next carried a similar epitasis analysis with *walrus* (*wal*). Remarkably, the combined expression of *wal*^RNAi^ and *osi*^RNAi^ resulted in a completely viable progeny, underscoring that Osi takes part in this crucial regulatory step (Figs. 7K and 12B).

Taken together, these results support the claim that Osi is the fly’s functional homolog of ETFRF1/LYRm5 and they reveal a new player in the regulation of lipid metabolism. On a greater note, Osi directly associates a cell-autonomous biochemical process with the overall growth of the cell and, in consequence, the organism, highlighting a potential communication between the fat body and the brain that results in a plastic food-seeking behavior.

## Discussion

In the search for genes essential for cellular homeostasis, we identified Orsai, a novel Complex I_LYR domain-containing protein. A severe loss of function allele or ubiquitous knockdown triggers developmental arrest at the L1 stage, followed by larval lethality. Depleted Orsai function is associated with exacerbated lipid catabolism, which in turn leads to the depletion of energy reserves which might cause the developmental arrest. Furthermore, mosaic analysis indicated that Orsai affects cell size in a cell-autonomous manner. Interestingly, in certain biological contexts (i.e., *osi* downregulation in the fat body), this essential function is fully rescued through the expression of human ETFRF1/LYRm5, strongly suggesting that ETFRF1/LYRm5 (Q6IPR1) is the human ortholog of CG6115/Osi (Q9VJG4).

### How does Osi contribute to cellular metabolism?

Figure 12 describes our working model regarding the contribution of Osi to the regulation of basal metabolism and the coordination of cell growth. During larval development, there is an increased demand for the activity of enzymes involved in glycolysis that promote carbohydrate metabolism, while β-oxidation of fatty acids is attenuated [12]. If Osi acts similarly as it has been shown in vitro for ETFRF1/LYRm5, it would inhibit ETF activity and block β-oxidation, favoring the accumulation of fatty acids required at later stages of development (i.e., during metamorphosis [43]). On the contrary, in the context of a severe Osi knockdown, an elevated ETF activity would be anticipated, resulting in an increase of β-oxidation. Interestingly, this scenario does not correlate with an increased electron flow to the ETC, as supported by the observation that *osi* mutants exhibit a reduced response to inhibitors of the respiratory chain (Fig. 8B). Instead, these electrons could input the reverse electron transfer (RET, see below), resulting in increased ROS levels (Fig. 8D) as well as an imbalance of intermediate metabolites (i.e., LCFAs) that could potentially affect membrane integrity and compromise cellular viability [44], as shown by the reduction of high molecular weight TAGs (Fig. 11B). Particularly, changes in the composition of the LD membrane might contribute to changes in their size distribution in *osi* mutants (Fig. 10G–I), which in turn could modulate the LD access to lipases, thereby increasing TAG degradation and fatty acid availability [45]; in line with this, downregulation of *Lip3* counteracts the active loss of energy reserves triggered by *osi* depletion (Fig. 9G–I), underscoring that phenotypes might be in part triggered by excessive lipid catabolism. In addition, increased ROS production as a result of a non-regulated ETF complex could lead to an increased lipid peroxidation, thereby affecting membrane fluidity, dynamics, transport eventually leading to defects in membrane initiated signaling [46].

#### A possible link to canonical pathways that regulate cell growth

Deregulated ETF/EtfQO activity results in higher free radical levels due to the exacerbated electron flow, as well as an increased fatty acid catabolism due to the inability to regulate this modulatory step (Fig. 8). In the context of an enhanced electron flow, the reduced state of coenzyme Q (CoQH2) would be increased, overwhelming its oxidative capacity, in turn promoting a retrograde flux of electrons (RET) from CoQH2 to Complex I (CI) [47]. As a consequence of this reversed electron transport, CI generates superoxide radicals and increases ROS production, as detected in *osi*-depleted larvae (Fig. 8). In this regard, the subtle rescue of the lethality by Sod2 is in agreement with earlier reports stating that Sod2 overexpression protects only weakly against ROS generated in CI, probably due to topological restrictions that limit proper access to those complexes [47, 48]. An additional consequence of RET is a decrease in NADH/FADH reducing agents that could, in turn, slow down glycolysis and the tricarboxylic acid cycle; in fact, *osi* mutants show reduced glycolysis (Fig. 8C). In addition, the unrestricted use of fatty acids depletes energy reserves, signaling nutritional stress, which could in time inhibit the classical cell growth regulatory pathway AKT/TOR and promote the degradation of more fatty acids in a futile attempt to maintain ATP levels [49–53]. Low ATP levels coupled to the generation of reactive oxygen species impact on the regulation of the TOR pathway further preventing cell growth in *osi* mutants [54].

### How are systemic effects generated?

Food-seeking behavior is precisely regulated by the integration of the internal energy status and external sensory signals. Upon starvation, larvae initiate a wandering-like behavior, the most prominent behavioral phenotype of *osi* mutants, probably in search for more nutritious food sources [55] (Fig. 1). Reducing Osi levels exclusively in the fat body is sufficient to trigger this behavior, in favor of a metabolic origin as opposed to one resulting from impaired Osi function in the brain. This behavioral response can then be reverted by ETFRF1/Lyrm5 expression in the fat body (Fig. 6B–E), further supporting its connection to lipid metabolism. In addition, *osi*-depleted animals exhibit increased *Lip3* levels (Fig. 9A, C), a marker of starvation [38], which could be the origin of the *orsai* (out of patch) phenotype.

Alteration of metabolic homeostasis correlates with changes in behavioral programs that in time lead to an adaptive feeding response [9, 56–59]. Previous studies have reported a similarly aberrant food-seeking behavior as the one displayed by *orsai* mutants, where altering fat body homeostasis eventually leads to the sudden and early cessation of feeding behavior [17, 55]. Even though there are many instances where signaling from the CNS leads to changes in peripheral tissues [9, 56, 60], and that communication between the fat body and the CNS has clearly been established [61, 62], no specific pathway has been associated to lipid metabolism. Thus, our results reveal a novel communication from the fat body to the brain and posit this model as an ideal one to reveal peripheral signals that modify behaviors.

## Conclusion

The data presented herein show that *osi* is a central player in lipid and energy metabolism and establish *osi* mutants as a suitable genetic model for further studies of conserved functions of the LYRm family of metabolic regulators.

## Methods

### Fly stocks and maintenance

Flies were grown and maintained in vials containing standard cornmeal medium at 25°C under 12:12 h light: dark cycles. The stocks used were *w*^1118^ (genetic control for transgenic flies), *act-*Gal4, *heat shock* (*hs*)-Flp, *elav*-Gal4 (8765), *pumpless*-Gal4 (*ppl*-Gal4), UAS-*sod2* (24494), UAS-*Lip3*^RNAi^ (65025), *osi*^Gal4^ (83190), UAS-CD8mCherry (27391), UAS-GFP (9431), UAS-*Dcr2* (24650), UAS-*wal*^RNAi^ (34915), and UAS-EtfQ0^RNAi^ (56864) which were obtained from the Bloomington Stock Center; UAS-*osi*^RNAi^ (construct ID 29711) was obtained from VDRC; and *nsyb*-Gal4, *cg*Gal4, and NP1Gal4 were donated by I. Miguel-Aliaga (Imperial College, UK), M. Katz (School of Medicine, UBA, Argentina), and A. Garelli (INIBBIB, Argentina), respectively. Unless otherwise indicated in the figure legend, a UAS*-dicer2* transgene was included in all RNAi experiments to bolster the effect of the UAS-*osi*^RNAi^. The *osi*^*100B/100B*^ mutation was backcrossed to *w*^1118^ for 5 generations. More details about stocks and antibodies used are included in Additional file 1: Supplementary Table 1.

### Sequence analysis

Protein sequence for CG6115 (Osi) of *Drosophila melanogaster* was obtained from the GeneBank database (NP_652578.1). Homology with different model organisms was tested by the Basic Local Alignment Search Tool (BLAST-p, NIH, https://blast.ncbi.nlm.nih.gov). Subsequently, each selected homolog was aligned using the ClustalX 2.1 alignment tool in order to identify the degree of similarity between the different organisms.

*Drosophila* and human sequences of the complex I LYR family (as defined by Uniprot) were retrieved. We retained for the analysis only reviewed human sequences as well as all *Drosophila melanogaster* entries. The resulting set has 11 human and 7 fly proteins. In fact, fly sequences were originally 8, but the unreviewed entry A0A0F6QCW0_DROME is identical to the reviewed one BCN92_DROME. All the proteins belong to the Pfam family PF05347 except for SDHF3_HUMAN and SDHF3_DROM of the PF01233 family. Both families are related and are part of the Complex I_LYR-like superfamily.

### Generation of transgenic lines

A Nt and Ct FLAG-tagged version of Osi (with a 6xGly linker between Osi and the Flag) and an untagged version of OSI were cloned into pUAStattB and targeted to the 86F8 recombination site on the 3rd chromosome. Additionally, a human ETFRF1/ LYRm5 and an *osi* sequence with changes in codon frequency in order to prevent silencing through the RNAi machinery (Osi-silent mutations- osi^SM^) were cloned into pUAStattB and targeted to the 28E7 recombination site on the 2nd chromosome.

For the generation of the pUAST-*osi*, pUAST-*osi*(flag) and pUAST-(flag)*osi* constructs, primers flanking the gene were used to amplify the desired sequence from larval cDNA and cloned in a pCR Blunt II-TOPO vector (450245, Thermo Fisher Scientific, USA) to be later subcloned in a pUASTattB vector (Catalog number 1419, Drosophila Genomics Resource Center, USA) by EcoR1 digestion sites. For the UAS-*osi* construct, the forward primer contained a *Drosophila* Kozak sequence before the ATG (Fw1), while the reverse primer contained a stop codon at the end (Fw1: 5′-GCCACCATGTCACAGCTGCGCTCGAAAG-3′; Rv1: 5′-TCAGTCATTGTAACTATAGCGCTGC-3′). In the UAS-*osi*(flag) construct, we used the same forward primer while the reverse was divided in two primers used in two consecutive PCR reactions with the insertion of a 6xGly linker (Rv2) followed by the FLAG (Rv3) sequence (Fw1: 5′-GCCACCATGTCACAGCTGCGCTCGAAAG-3′,Rv2: 5′-GTAATCTCCACCCCCGCCTCCCCCGTCATTGTAACTATAGCGCTGC-3′, Rv3: 5′-TCACTTATCATCATCATCCTTGTAATCTCCACCCCCGCCTCCCCC-3′). Similarly, the UAS-(flag)*osi* construct was designed to carry a 6xGly linker (Fw2)) and a FLAG following the kozak sequence (Fw3) in the forward primer, while the reverse (Rv3) one included a stop codon (Fw2: 5′-TAAGGGGGGAGGCGGGGGTGGATCACAGCTGCGCTCGAAAG-3′, Fw3: 5′-GCCACCATGGATTACAAGGATGATGATGATAAGGGGGGAGGCGGGGGTG-3′, Rv3: 5′-TCAGTCATTGTAACTATAGCGCTGC-3′).

The *Drosophila* codon-optimized version of human ETFRF1/LYRm5 and the *osi*^SM^ constructs were obtained from GeneScript (USA) and cloned separately in the pUASTattB vector by the EcoR1 and Xba1 digestion sites. A comparison of the original and codon-optimized sequences is detailed in Additional file 1: Fig. S1 in. Injections and selection of transgenic individuals were carried out by Best Gene (USA).

### Growth and survival curves

For growth assessment, ten larvae per genotype aged 24 h AEL were placed in standard agar plates (3% sucrose). Every 24h, larvae still alive were imaged to assess growth by measuring body size (see below) and transferred to a new plate. Experiments lasted as long as any mutant or RNAi-expressing individuals could be scored as alive.

Survival rates were examined taking 50 larvae per genotype 24h AEL that were placed in standard agar plates. Every 24h, those that were still alive were transferred into a new plate containing a yeast patch until adult eclosion or until the death of Osi-depleted animals.

### Larval morphology assessment

To compare body size between larvae of different genotypes or experimental conditions, we used an Olympus DP71 camera attached to an Olympus BMX10 stereoscope to take pictures of the larvae at 24-h intervals starting with larvae aged 24h AEL. ImageJ was used to measure the area of the image occupied by the larva’s entire body. At the end of the experiment, the sclerotized mouth parts (“hooks”) were dissected and photographed for larval staging (employing an Olympus BX60 microscope).

### Colored food assay

Young (7-day old) female flies were allowed to lay eggs for 4h. Twenty-five larvae of the corresponding age of each genotype were transferred to separate agar chambers with a patch of yeast paste (food) mixed with commercial blue food coloring. One hour later, the numbers of larvae inside or outside the colored food patch were recorded. Individual larvae were also photographed as described above. Larvae that did not display a colored intestine were quantified as “clear gut” larvae.

### Two-choice olfactory assay

An olfactory attractive response was measured as described previously [22]. Groups of fifty 24h AEL larvae were placed in the center of an agar plate, where filter papers of 5 mm of diameter, soaked with an attractant (propionic acid) or a neutral compound (water), were placed on opposite sides of the dish. Larvae were photographed right after they were placed on the plate (initial time) and 5 min later (final time). We calculated the Response Index, defined as:

$$RI=\frac{Na- Nn}{Na+ Nn}$$

where RI = response index, Na = number of larvae under 30 mm from the attractive stimulus, and Nn = number of larvae under 30 mm from the neutral stimulus. Positive RI represents attraction, while negative RI represents avoidance and RI near zero represents indifference. Each experimental group was tested 3 times.

### Mitochondrial extracts and functional assessments

Mitochondrial extracts were obtained from 5 mg of either control (*w*^1118^) or homozygous *osi*^100B^ 72h AEL larvae using the Mitochondria Isolation Kit for Tissue (89801, Thermo Fisher Scientific, USA) and the Dounce Homogenization for Hard Tissue protocol with trypsin pre-treatment. We modified the protocol and used only half of the volume of the different solutions needed. For protein quantification, we used the mitochondria lysis protocol as the manufacturer instructed, followed by the Pierce BCA Protein Assay (23227, Thermo Fisher Scientific, USA). Measurements were made using a microplate reader. From these samples, mitochondrial ATP production was measured with a commercial kit (Adenosine 5’triphosphate (ATO) bioluminescent assay kit; FLAA-1KT, Sigma-Aldrich, USA) according to the manufacturer’s instructions. The experiment was performed 3 times independently.

Superoxide levels were assessed according to Owusu-Ansah and Banerjee as modified by Lim et al. [34, 63]. In the final step, glycerol was used instead of the ProLong Gold antifade reagent. Two independent assays were performed.

### Quantitative real-time reverse transcriptase (RT) PCR (qPCR)

Twenty to thirty larvae (96 and 72h AEL respectively) were collected per replicate and homogenized with an Omni bead ruptor IV (USA) in 500 μl Trizol reagent (15596026, Thermo Fisher Scientific, USA) according to the manufacturer’s instructions in order to obtain total RNA. Next, synthesis of cDNA was performed from 1 μg of total RNA with the SuperScript III Reverse Transcriptase kit (18080093, Thermo Fisher Scientific, USA) according to the manufacturer’s instructions employing oligo(dt) and gene-specific primers for *tweek*, *Lip3*, *Pepck1*. qPCR was performed on a Stratagene Mx3000P (Agilent Technologies) using FastStart Universal SYBR Green Master (04913914001, Roche, USA) in a 10 μl reaction. The PCR reaction consisted of 40 cycles of a 15-s denaturation step at 95°C, a 15-s hybridization step at 60°C, and a 30-s extension step at 72°C. A minimum of three independently collected biological replicates were used in each experiment and the data was expressed as the ratio of each specific gene over *Rpl29*. The following primers were used: *rpl29* (fw 5′GAACAAGAAGGCCCATCGTA3′; rev 5′AGTAACAGGCTTTGGCTTGC3′); *Lip3* (fw 5′TTCTTCCTCCGATTGGGTGCTCAT3′; rev 5′AACGTCGTACATGCCGATCTCGTT3′); *Pepck* 1(fw 5′AGAAGAAGTACATCACTGCCGCCT3′; rev 5′TCCCTGCGAGTCAAACTTCATCCA3′); *tweek* (fw 5′GTGGATGTACCCTATGCCCG3′; rev 5′TCTTAGGTGGTGAAACCCGC3′); *osi* (fw 5′ACAAACCAATTCCGCTGACCACTG3′; rev 5′ATCCTGCTCGTCCTTGTGGTTCAT3′).

### Dietary supplementation of NAC

Antioxidant dietary supplementation was achieved by addition of 0.8 mg/ml N-acetyl-cysteine (NAC, Sigma-Aldrich). Twenty to thirty 24h AEL larvae were selected and placed in vials with normal food supplemented with NAC according to [64]. Survival was assessed every 24h. Three replicates of each genotype per experiment were carried out; two independent experiments were performed.

### Immunohistochemistry

Larvae were dissected in dissection buffer (70mM Na_2_HPO_4_, 30mM Na_2_HPO_4_, 0,15 M NaCl, 0.3% Triton X-100, pH 7,4, -PT-) and fixed in 4% formaldehyde pH 7.4 in 100 mM PBS 1× for 45 min at RT. Larvae were rinsed three times in PT for 5 min. Non-specific binding sites were blocked by incubating the tissues for 30 min in 10% goat serum in PT. The tissues were incubated in the corresponding primary antiserum at 4°C overnight. Primary antibodies were as follows: chicken anti-RFP (1/500 Rockland, USA), chicken anti-GFP (1/500 Aves, USA), and monoclonal anti Flag (1/500 Thermo Scientific, USA). Samples were washed 3 times in PT 0.3% for 5 min and incubated with the corresponding secondary antibodies (Cy2, Cy3- anti chicken, Cy5- anti mouse, Jackson ImmunoResearch) for 2h at RT. Secondary antibody incubations were stopped by replacing the solution with PT 3 times for 15 min each. For visualization of lipid droplets, 24, 48, and 72h AEL larvae were dissected as previously described and fixed in 4% formaldehyde pH 7.4 in 100 mM PBS 1× for 60 min at RT. Tissues were then incubated in BODIPY (1:500 D3922, Thermo Fisher Scientific) for 20 min at RT. Samples were then washed 3 times in PT 0.3% for 5 min and mounted.

If needed, samples were incubated with DAPI 1× and/or rhodamine-coupled Phalloidin 1× for 30 min and then rinsed 3 times for 5 min in PT. Finally, samples were mounted in 60% glycerol in PT. Images were taken with a Zeiss confocal microscope LSM 710.

### Mosaic analysis in the fat body

The “Flip-out” technique (reviewed in [65]) was employed to investigate the effect of Osi loss of function in cell size (in the genotype hs-*flip*; act>STP>G4;UAS-*gfp*/UAS-*osi*^RNAi^). A 5-min 37°C heat pulse in a water bath was used to activate the heat-shock flipase (*hs*-flip) in 24h AEL larvae, which in turn removed the stop cassette from an *act*-G4 driver, inducing the expression of green fluorescence marker (GFP) along with the construct of interest in a subset of cells. Fat bodies from 72h post heat-shock larvae were dissected and stained for GFP (as detailed above) and phalloidin red (1:100, R415, Sigma-Aldrich, USA) to mark cellular boundaries.

Both control (GFP−) and experimental (GFP+) cells, expressing the construct of interest, were measured using ImageJ. Measurements were normalized to the average cell size of control cells (inactive). We used R-studio to plot frequency distribution graphs for each genotype using function

- *hist(data$genotype-activationstate,xlim=range(0:2), ylim=range(0:4), prob=TRUE)*

We dissected 3 independent larvae of each genotype. Images were taken with a Zeiss LSM 710 confocal microscope. We quantified cellular area using ImageJ.

### Quantitative analysis of lipid droplets

To assess lipid droplets size and shape 24, 48, and 72h AEL, larval fat bodies were dissected, fixed, and stained with BODIPY. Images were taken with a Zeiss LSM 880 confocal microscope. Each image was assigned a random numeric code in order to quantify the different aspects of each image in blind. To standardize the measurements among the different groups, we randomly selected a representative squared area (50μm) per image, to which all measurements were then normalized. The area span by individual lipid droplet, their roundness and percentage of reactive area were measured with ImageJ as previously described [66, 67]. Briefly, LD were delimited by free hand with the selection tool and then the area and roundness were assessed; to define the percentage of reactive area the threshold tool was employed to generate a mask representing the total area of the fat body occupied by LDs. GraphPad Prism 9.0 was used to plot and quantify the data and to construct the LD size distribution histograms.

### Respiratory assays

The Agilent Seahorse XFp metabolic analyzer was set to a working temperature of 25°C. An Agilent Seahorse XFp cartridge (Agilent, 103721-100) was hydrated with 200 μl of calibrant solution (Agilent, 100840-000) overnight at 25°C. The next day, 20 larvae (72h AEL) for each well were dissected in phosphate buffered solution (PBS) and added to the bottom of an Agilent 8-well cell plate (Agilent, 103721-100), centered in the middle between the three raised spheres, with the help of a drop of Vetbond (3M) tissue adhesive to keep them in place. Each well was then filled with 50 μl of AHL for the mitostress and 50 μl of Agilent Seahorse assay media for the gluco-stress with the corresponding supplements required for the specific assays. Then, 150 μl of assay media was added to each well, resulting in a total of 200 μl final in each well. Cell plate was then placed on the tray of the XFp analyzer. The instrument was used as is for typical cell assays with all cycle procedures consisting of 3 min mixing, 0 min waiting, and 3 min measuring.

#### Mitostress assay

Analysis of mitochondrial respiration was conducted in Agilent Seahorse XF AHL medium (*a*dult *h*emolymph like *s*aline containing 108 mM NaCl, 5 mM KCl, 2 mM CaCl2, 8.2 mM MgCl2, 4 mM NaHCO3, 1 mM NaH2PO4, 5 mM trehalose, 10 mM sucrose, 5 mM HEPES, pH 7.5, [68]). Basal larvae OCR was measured for 3 cycles prior to oligomycin injections. Twenty-five microliters of 100 μM oligomycin was added to injection port A, resulting in a final concentration of 10 μM of oligomycin/ well. Twenty-five microliters of 70 μM Carbonyl cyanide-4 (trifluoromethoxy) phenylhydrazone (FCCP) was added to port B and injected after the 6th cycle, resulting in a final concentration of 7 μM FCCP. Twenty-five microliters of 100 μM Rotenone/Antimycin A were added to port C and injected after the 9th cycle, resulting in a final concentration of 10 μM Rotenone/Antimycin A.

#### Glycostress assay

Analysis of larval glycolytic function, by directly measuring the extracellular acidification rate (ECAR), was conducted in base medium (Agilent, 103334-100 base medium) supplemented with 1 mM glutamine. Samples were starved for 60 min prior to testing. Twenty-five microliters of 50 mM glucose was added to port A and injected at the 6th cycle, resulting in a final concentration of 5 mM glucose. Twenty-five microliters of 100 μM oligomycin was added to port B and injected at the 11th cycle, resulting in a final concentration of 10 μM oligomycin. Twenty-five microliters of 1 M 2- deoxyglutarate (2-DG) was added to port C and injected at the 23rd cycle, resulting in 100 mM 2-DG.

### Lipidome analysis

All solvents were at least HPLC grade. Water, 2-propanol, and phosphoric acid were purchased from Roth (Karlsruhe, GER), methanol from J.T.Baker (Austin, TX, USA), formic acid, TAG 45:0 and TAG 51:0 from Sigma (Vienna, AUT), and ammonium acetate from Merck (Darmstadt, GER). Glass beads (0.45–0.50 mm) were from SiLi (Warmensteinach, GER).

#### Sample preparation

Approximately 50 μl of glass beads and 400 μl of isopropanol were added to 50 frozen larvae in the Eppendorf tubes. The larvae were disrupted by shaking the tubes at 4°C in a MM 40 homogenizer (Retsch, Haan, GER) for 20 min at 30 Hz. Lipid extraction was done according to the method described by [69]. In brief: The isopropanol was removed in a speed vac and 700 μl MTBE/methanol (10/3, v/v) containing TAG 45:0 and TAG 51:0 internal standards. After shaking at 4°C for 60 min with a Thermomixer (Eppendorf), 400 μl water was added and shaking continued for another 15 min. After centrifugation (15 min, 13,000 rpm), 50 μl of the organic upper phase was transferred to an autosampler vial, dried under a stream of nitrogen and re-dissolved in 400 μl isopropanol/methanol/water (30/15/5, v/v/v) for LC-MS analysis. The remaining organic upper phase (approximately 350 μl) was collected, dried under nitrogen and stored at −80°C.

#### Protein analysis

The remaining aqueous phase was dried in a speed vac. Then, 400 μl of 0.1 N NaOH were added, and after shaking with the Retsch Mill (10min at 30 Hz), the samples were incubated for 6 h at 60°C. Twenty microliters was used for protein analysis with the Pierce™ BCA protein assay kit (Thermo Fisher, Vienna, AUT).

#### Lipid analysis

Chromatographic separation was performed using a 1290-UHPLC system (Agilent, Waldbronn, GER) equipped with a BEH-C18-column, 2.1 × 150 mm, 1.7 μm (Waters, Manchester, UK). The autosampler compartment was set to 8°C and 1 μl sample was injected. A binary gradient was applied. Solvent A was water, solvent B was 2-propanol. Both solvents contained phosphoric acid (8 μM), ammonium acetate (10 mM), and formic acid (0.1 vol%). The linear gradient started at 50% solvent B at a constant flow rate of 0.15 ml/min and increased to 100% solvent B within 22 min. In the following, 2.5 min solvent B percentage was kept at 100%. The column was re-equilibrated for 5 min, resulting in a total HPLC run time of 30 min. The column compartment was kept at 50°C. A 4670 triple quadrupole mass spectrometer (Agilent) equipped with an ESI source was used for analysis. The following source parameters were used: source temperature 300°C, sheath gas (N2) temperature 400°C. The capillary voltage was 3.5 kV in positive ionization mode. Samples were analyzed in positive ionization mode via dynamic MRM scans (For a detailed MRM list see Additional file 1: Supplementary Table 2). Data analysis was done using the MassHunter 10.0 software package (Agilent). Peak areas were normalized both to internal standard peak areas and to protein amount of sample.

In Fig. 11A, one of the three values obtained for osi^100B^/+ at 72h was excluded from statistical analysis (all datasets are included in Additional file 2). This value was excluded since it was one order of magnitude below the other two replicas.

### Statistical analysis

Statistical analyses were performed with R-Studio (Free Software Foundation, Inc) and GraphPad Prism 9.0 (GraphPad Software, Inc). In all graphs, results are presented as mean ± SEM, and experimental groups labeled with different letters that indicate statistically significant differences, with a *p* < 0.05. For every experiment, statistical tests are stated in each figure, for detailed information see Additional file 2. Unless otherwise stated, each experiment consisted of at least 3 independent replicates.

## Supplementary Information

**Additional file 1: Table S1.** List of fly stocks, antibodies and fluorescent dyes used throughout this study. **Figure S1.** Sequence comparison of *osi*^SM^ and ETFRF1/LYRm5. Alignment of the original ETFRF1 and *osi* mRNA sequences, compared to the version optimized for expression in *Drosophila*. **Table S2**. List of dynamic MRM transitions. it includes the list of dynamic MRM transitions.

**Additional file 2 **It includes all datasets and statistical analysis on which the conclusions are based. **Figure 1**. P[UAS] insertional mutant exhibits abnormal food-seeking behavior. **Figure 2**. P[UAS]100B is inserted in CG6115 and encodes a Complex I LYR domain containing protein. **Figure 3**. A reduction in osi levels affects viability. **Figure 4**. osi downregulation is associated with a smaller cell size. **Figure 5**. ETFRF1/LYRm5 rescues cellular and viability defects associated with osi knockdown. **Figure 6**. Expression of human ETFRF1/LYRm5 rescues lethality associated with osi downregulation in the fat body. **Figure S7**. A key role for osi in the fat body. **Figure 8**. osi loss of function produces a metabolic defect not rescued by antioxidants. **Figure 9**. Preventing excessive fat body lipid catabolism rescues the behavioral effect triggered by osi downregulation. **Figure 10**. osi mutants have a progressive shift in LD size. **Figure 11**. Reduced osi levels alters the storage lipid content and composition. **Figure 12**. A model for Osi function.

## Data Availability

All data generated or analyzed during this study are included in this published article and its supplementary information files. Any other data can be requested from the corresponding author.
