# Supplementary material for: orsai, the Drosophila homolog of human ETFRF1, links lipid catabolism to growth control
Source: BMC Biol. 2022 Oct 21;20:233. doi: 10.1186/s12915-022-01417-w (PMC9585818; doi:10.1186/s12915-022-01417-w)
Supplement: Supplementary file 1 — Additional file 1: Table S1. List of fly stocks, antibodies and fluorescent dyes used throughout this study. Figure S1. Sequence comparison of osiSM and ETFRF1/LYRm5. Alignment of the original ETFRF1 and osi mRNA sequences, compared to the version optimized for expression in Drosophila. Table S2. List of dynamic MRM transitions. it includes the list of dynamic MRM transitions. [file 12915_2022_1417_MOESM1_ESM.docx]

**Additional File 1**

**Table** S**1**. List of fly stocks, antibodies and fluorescent dyes used throughout this study.

| **Lines** | **Identifier for publication** | **Number of Stock** | **Stock Center** |
| --- | --- | --- | --- |
| w1118 | RRID:BDSC_5905 | 5905 | Bloomington Stock Center |
| act-Gal4 | RRID:BDSC_4414 | 4414 | Bloomington Stock Center |
| heat shock (hs)-FLP | RRID:BDSC_6 | 6 | Bloomington Stock Center |
| nSyb-Gal4 | Facilitated by Irene Miguel Aliaga | | |
| elav-Gal4 | BDSC_8765 | 8765 | Bloomington Stock Center |
| pumpless-Gal4 (ppl-Gal4) | RRID:BDSC_58768 | 58768 | Bloomington Stock Center |
| UAS- sod2 | RRID:BDSC_24494 | 24494 | Bloomington Stock Center |
| UAS-Lip3RNAi | RRID:BDSC_65025 | 65025 | Bloomington Stock Center |
| osi-Gal4 | RRID:BDSC_83190 | 83190 | Bloomington Stock Center |
| UAS -CD8mCherry | RRID:BDSC_27391 | 27391 | Bloomington Stock Center |
| UAS-GFP | from line RRID:BDSC_9431 | 9431 | Bloomington Stock Center |
| UAS-Dcr2 | RRID:BDSC_24650 | 24650 | Bloomington Stock Center |
| UAS -walRNAI | RRID:BDSC_34915 | 34915 | Bloomington Stock Center |
| UAS -EtfQ0RNAi | RRID:BDSC_56864 | 56864 | Bloomington Stock Center |
| UAS-osiRNAi | Construct ID 15140 | 29711 | VDRC |
| cg-Gal4 | - | - | Donated by M. Katz |
| NP1-Gal4 | - | - | Donated by A. Garelli |
| osi100B/100B | Generated in the laboratory, please contact MF Ceriani | | |
| UAS-osi(flag) | Generated in the laboratory, please contact MF Ceriani | | |
|  |  |  |  |
| **Antibodies** | **Manufacturer** | **Catalog number** | **RRID** |
| Anti-RFP (chicken) | Rockland, PA | 600-901-379S, | AB_10703148 |
| Anti-GFP (chicken) | Aves, Oregon | GFP-1020 | AB_10000240 |
| Anti Flag monoclonal (mouse, IgG2b) | Thermo Scientific, USA | FG4R | AB_1957945 |
| DyLight488-conjugated anti chicken (donkey) | Jackson Immunoresearch | 703-485-155 | AB_2340375 |
| Cy3-conjugated anti chicken (donkey) | Jackson Immunoresearch | 703-165-155 | AB_2340363 |
| Cy 5-conjugated anti mouse (donkey) | Jackson Immunoresearch | 715-175-150 | AB_2340819 |
|  |  |  |  |
| BODIPY™ 493/503 | Thermo Fisher Scientific, USA | D3922 | Not available |
| Rhodamine conjugated phalloidin | Invitrogen/ Thermo Fisher Scientific, USA | R415 | AB_2572408 |

**Fig S1.** **Sequence comparison of *osi*^SM^ and ETFRF1/LYRm5**. Alignment of the original ETFRF1 and *osi* mRNA sequences (top sequences in **A** and **B**, respectively) compared to the version optimized for expression in *Drosophila* (Bottom sequences). No changes to the coding sequence were introduced. In the case of Osi, silence mutations were introduced to avoid recognition by the expressed RNAi construct. In red, the designed sequence to be targeted by the RNAi machinery.

**Table** S**2**. List of dynamic MRM transitions

| **Cpd Name** | **Prec Ion** | **Prod Ion** | **Frag**  **(V)** | **CE**  **(V)** | **Cell Acc (V)** | **Ret Time (min)** | **Ret Window**  **(min)** | **Polarity** |
| --- | --- | --- | --- | --- | --- | --- | --- | --- |
| DAG 24:0 -12:0 | 474.4 | 257.3 | 110 | 16 | 5 | 12 | 9 | + |
| DAG 25:0 -12:0 | 488.4 | 271.3 | 110 | 16 | 5 | 12 | 9 | + |
| DAG 26:0 -14:0 | 502.4 | 257.3 | 110 | 16 | 5 | 12 | 9 | + |
| DAG 26:1 -14:0 | 500.4 | 255.3 | 110 | 16 | 5 | 12 | 9 | + |
| DAG 27:0 -14:0 | 516.5 | 271.3 | 110 | 16 | 5 | 14.3 | 9 | + |
| DAG 28:1 -14:0 | 528.5 | 283.4 | 110 | 16 | 5 | 13.7 | 9 | + |
| DAG 29:0 -14:0 | 544.5 | 299.4 | 110 | 16 | 5 | 14.5 | 9 | + |
| DAG 30:0 -16:0 | 558.5 | 285.4 | 110 | 16 | 5 | 14 | 9 | + |
| DAG 30:1 -16:0 | 556.5 | 283.4 | 110 | 16 | 5 | 13.37 | 9 | + |
| DAG 30:2 -16:1 | 554.5 | 283.4 | 110 | 16 | 5 | 15 | 9 | + |
| DAG 31:0 -16:0 | 572.5 | 299.4 | 110 | 16 | 5 | 14.5 | 9 | + |
| DAG 32:0 -16:0 | 586.5 | 313.4 | 110 | 16 | 5 | 14.1 | 9 | + |
| DAG 32:1 -16:1 | 584.5 | 313.4 | 110 | 16 | 5 | 13.4 | 9 | + |
| DAG 32:2 -16:1 | 582.5 | 311.4 | 110 | 16 | 5 | 12.7 | 9 | + |
| DAG 32:3 -16:1 | 580.5 | 309.4 | 110 | 16 | 5 | 12.7 | 9 | + |
| DAG 33:0 -16:0 | 600.6 | 327.5 | 110 | 16 | 5 | 15.2 | 9 | + |
| DAG 34:0 -16:0 | 614.6 | 341.5 | 110 | 16 | 5 | 14 | 9 | + |
| DAG 34:1 -16:1 | 612.6 | 341.5 | 110 | 16 | 5 | 13.5 | 9 | + |
| DAG 34:2 -16:1 | 610.5 | 339.4 | 110 | 16 | 5 | 13 | 9 | + |
| DAG 34:3 -16:1 | 608.5 | 337.3 | 110 | 16 | 5 | 15 | 9 | + |
| DAG 36:1 -18:0 | 640.6 | 339.4 | 110 | 16 | 5 | 15 | 9 | + |
| DAG 36:2 -18:1 | 638.6 | 339.4 | 110 | 16 | 5 | 15 | 9 | + |
| DAG 36:3 -18:1 | 636.6 | 337.3 | 110 | 16 | 5 | 15 | 9 | + |
| DAG 36:4 -18:2 | 634.5 | 337.3 | 110 | 16 | 5 | 15 | 9 | + |
| IS LPC 17:0 | 510.3 | 184.1 | 179 | 28 | 5 | 7.4 | 9 | + |
| IS LPC 17:1 | 508.3 | 184.1 | 79 | 28 | 5 | 6.4 | 9 | + |
| IS LPG 17:1 | 466.3 | 325.2 | 111 | 16 | 5 | 6.7 | 9 | + |
| IS PC36:0 d68 | 858.6 | 184.1 | 164 | 28 | 5 | 14.4 | 9 | + |
| IS PC36:0 d70 | 860.5 | 184.1 | 164 | 28 | 5 | 14.4 | 9 | + |
| IS PE 34:0 | 720.6 | 579.5 | 131 | 20 | 5 | 13.55 | 9 | + |
| IS TG 45:0 | 782.7 | 523.5 | 169 | 24 | 5 | 20 | 9 | + |
| IS TG 51:0 | 866.8 | 579.5 | 179 | 24 | 5 | 21.51 | 9 | + |
| LPC 14:0 | 468.3 | 184.1 | 179 | 28 | 5 | 5 | 9 | + |
| LPC 16:0 | 496.3 | 184.1 | 179 | 28 | 5 | 8 | 9 | + |
| LPC 16:1 | 494.3 | 184.1 | 179 | 28 | 5 | 7.5 | 9 | + |
| LPC 18:0 | 524.3 | 184.1 | 179 | 28 | 5 | 8 | 9 | + |
| LPC 18:1 | 522.3 | 184.1 | 179 | 28 | 5 | 7 | 9 | + |
| LPC 18:2 | 520.3 | 184.1 | 179 | 28 | 5 | 6 | 9 | + |
| LPC 18:3 | 518.3 | 184.1 | 179 | 28 | 5 | 4.7 | 9 | + |
| LPE 16:0 | 454.3 | 313.3 | 111 | 16 | 5 | 6 | 9 | + |
| LPE 16:1 | 452.3 | 311.3 | 111 | 16 | 5 | 6 | 9 | + |
| LPE 18:0 | 482.3 | 341.3 | 111 | 16 | 5 | 6.4 | 9 | + |
| LPE 18:1 | 480.3 | 339.3 | 111 | 16 | 5 | 5.5 | 9 | + |
| LPE 18:2 | 478.3 | 337.3 | 111 | 16 | 5 | 4.8 | 9 | + |
| PC 26:0 | 650.5 | 184.1 | 180 | 30 | 5 | 10 | 9 | + |
| PC 27:0 | 664.5 | 184.1 | 180 | 30 | 5 | 11 | 9 | + |
| PC 28:0 | 678.6 | 184.1 | 180 | 30 | 5 | 11 | 9 | + |
| PC 28:1 | 676.6 | 184.1 | 180 | 30 | 5 | 11 | 9 | + |
| PC 28:2 | 674.6 | 184.1 | 180 | 30 | 5 | 11 | 9 | + |
| PC 29:0 | 692.5 | 184.1 | 180 | 30 | 5 | 12 | 9 | + |
| PC 29:1 | 690.5 | 184.1 | 180 | 30 | 5 | 12 | 9 | + |
| PC 30:0 | 706.6 | 184.1 | 180 | 30 | 5 | 12 | 9 | + |
| PC 30:1 | 704.6 | 184.1 | 180 | 30 | 5 | 12 | 9 | + |
| PC 30:2 | 702.6 | 184.1 | 180 | 30 | 5 | 12 | 9 | + |
| PC 32:0 | 734.6 | 184.1 | 180 | 30 | 5 | 12 | 9 | + |
| PC 32:1 | 732.6 | 184.1 | 180 | 30 | 5 | 12 | 9 | + |
| PC 32:2 | 730.6 | 184.1 | 180 | 30 | 5 | 12 | 9 | + |
| PC 32:2 | 728.6 | 184.1 | 180 | 30 | 5 | 12 | 9 | + |
| PC 34:0 | 762.6 | 184.1 | 180 | 30 | 5 | 13 | 9 | + |
| PC 34:1 | 760.6 | 184.1 | 180 | 30 | 5 | 11.8 | 9 | + |
| PC 34:2 | 758.6 | 184.1 | 180 | 30 | 5 | 11.1 | 9 | + |
| PC 34:3 | 756.6 | 184.1 | 180 | 30 | 5 | 11.1 | 9 | + |
| PC 34:4 | 754.6 | 184.1 | 180 | 30 | 5 | 11.1 | 9 | + |
| PC 34:5 | 752.6 | 184.1 | 180 | 30 | 5 | 11.1 | 9 | + |
| PC 36:0 | 790.6 | 184.1 | 180 | 30 | 5 | 13.5 | 9 | + |
| PC 36:1 | 788.6 | 184.1 | 180 | 30 | 5 | 12.8 | 9 | + |
| PC 36:2 | 786.6 | 184.1 | 180 | 30 | 5 | 13 | 9 | + |
| PC 36:3 | 784.6 | 184.1 | 180 | 30 | 5 | 13 | 9 | + |
| PC 36:4 | 782.6 | 184.1 | 180 | 30 | 5 | 13 | 9 | + |
| PC 36:5 | 780.6 | 184.1 | 180 | 30 | 5 | 13 | 9 | + |
| PC 36:6 | 778.6 | 184.1 | 180 | 30 | 5 | 13 | 9 | + |
| PC 38:0 | 818.6 | 184.1 | 180 | 30 | 5 | 13 | 9 | + |
| PC 38:1 | 816.6 | 184.1 | 180 | 30 | 5 | 13 | 9 | + |
| PC 38:2 | 814.6 | 184.1 | 180 | 30 | 5 | 13 | 9 | + |
| PC 38:3 | 812.6 | 184.1 | 180 | 30 | 5 | 13 | 9 | + |
| PC 38:4 | 810.6 | 184.1 | 180 | 30 | 5 | 13 | 9 | + |
| PC 40:0 | 846.7 | 184.1 | 180 | 30 | 5 | 13 | 9 | + |
| PC 40:1 | 844.7 | 184.1 | 180 | 30 | 5 | 13 | 9 | + |
| PE 26:0 | 608.4 | 467.4 | 131 | 20 | 5 | 10.5 | 9 | + |
| PE 28:0 | 636.5 | 495.5 | 131 | 20 | 5 | 10.5 | 9 | + |
| PE 28:1 | 634.5 | 493.5 | 131 | 20 | 5 | 10.5 | 9 | + |
| PE 29:0 | 650.5 | 509.5 | 131 | 20 | 5 | 11 | 9 | + |
| PE 30:0 | 664.5 | 523.5 | 131 | 20 | 5 | 11 | 9 | + |
| PE 30:1 | 662.5 | 525.5 | 131 | 20 | 5 | 11.5 | 9 | + |
| PE 31:0 | 678.5 | 537.5 | 131 | 20 | 5 | 13 | 9 | + |
| PE 31:1 | 676.5 | 535.5 | 131 | 20 | 5 | 13 | 9 | + |
| PE 32:0 | 692.5 | 551.5 | 131 | 20 | 5 | 13 | 9 | + |
| PE 32:1 | 690.5 | 549.5 | 131 | 20 | 5 | 12.4 | 9 | + |
| PE 32:2 | 688.5 | 547.5 | 131 | 20 | 5 | 12.4 | 9 | + |
| PE 32:3 | 686.5 | 545.5 | 131 | 20 | 5 | 12.4 | 9 | + |
| PE 33:0 | 706.5 | 565.5 | 131 | 20 | 5 | 12.1 | 9 | + |
| PE 33:1 | 704.5 | 563.5 | 131 | 20 | 5 | 12.1 | 9 | + |
| PE 34:0 | 720.5 | 579.5 | 131 | 20 | 5 | 12.1 | 9 | + |
| PE 34:1 | 718.5 | 577.5 | 131 | 20 | 5 | 12.1 | 9 | + |
| PE 34:2 | 716.5 | 575.5 | 131 | 20 | 5 | 11.5 | 9 | + |
| PE 34:3 | 714.5 | 573.5 | 131 | 20 | 5 | 11.5 | 9 | + |
| PE 34:4 | 712.5 | 571.5 | 131 | 20 | 5 | 11.5 | 9 | + |
| PE 35:0 | 746.6 | 605.6 | 131 | 20 | 5 | 13.1 | 9 | + |
| PE 35:1 | 734.6 | 593.6 | 131 | 20 | 5 | 13.1 | 9 | + |
| PE 36:0 | 748.6 | 607.6 | 131 | 20 | 5 | 13.1 | 9 | + |
| PE 36:1 | 746.6 | 605.6 | 131 | 20 | 5 | 13.1 | 9 | + |
| PE 36:2 | 744.6 | 603.6 | 131 | 20 | 5 | 12.5 | 9 | + |
| PE 36:3 | 742.6 | 601.6 | 131 | 20 | 5 | 11.6 | 9 | + |
| PE 36:4 | 740.6 | 599.6 | 131 | 20 | 5 | 11.4 | 9 | + |
| PE 36:5 | 738.6 | 597.6 | 131 | 20 | 5 | 11.4 | 9 | + |
| PE 36:6 | 736.6 | 595.6 | 131 | 20 | 5 | 11.4 | 9 | + |
| PE 38:0 | 776.6 | 635.6 | 131 | 20 | 5 | 12.5 | 9 | + |
| PE 38:1 | 774.6 | 633.6 | 131 | 20 | 5 | 11.5 | 9 | + |
| PE 38:2 | 772.6 | 631.6 | 131 | 20 | 5 | 11.3 | 9 | + |
| PE 38:3 | 770.6 | 629.6 | 131 | 20 | 5 | 11.3 | 9 | + |
| PS 34:0 | 764.5 | 579.5 | 131 | 20 | 5 | 11.9 | 9 | + |
| PS 34:1 | 762.5 | 577.5 | 131 | 20 | 5 | 11.9 | 9 | + |
| PS 34:2 | 760.5 | 575.5 | 131 | 20 | 5 | 11.9 | 9 | + |
| PS 34:3 | 758.5 | 573.5 | 131 | 20 | 5 | 11.9 | 9 | + |
| PS 36:1 | 790.6 | 605.6 | 131 | 20 | 5 | 11.7 | 9 | + |
| PS 36:2 | 788.6 | 603.6 | 131 | 20 | 5 | 11 | 9 | + |
| PS 36:3 | 786.6 | 601.6 | 131 | 20 | 5 | 11 | 9 | + |
| PS 36:4 | 784.6 | 599.6 | 131 | 20 | 5 | 11 | 9 | + |
| PS 36:5 | 782.6 | 597.6 | 131 | 20 | 5 | 11 | 9 | + |
| TAG 36:0-12:0 | 656.6 | 439.5 | 179 | 24 | 5 | 14 | 9 | + |
| TAG 36:1-12:0 | 654.6 | 437.5 | 179 | 24 | 5 | 14 | 9 | + |
| TAG 37:0-12:0 | 670.6 | 453.5 | 179 | 24 | 5 | 14 | 9 | + |
| TAG 37:1-12:0 | 668.6 | 451.5 | 179 | 24 | 5 | 14 | 9 | + |
| TAG 38:0-12:0 | 684.6 | 467.5 | 179 | 24 | 5 | 14 | 9 | + |
| TAG 38:1-12:0 | 682.6 | 465.5 | 179 | 24 | 5 | 14 | 9 | + |
| TAG 38:2-12:0 | 680.6 | 463.5 | 179 | 24 | 5 | 14 | 9 | + |
| TAG 39:0-12:0 | 698.6 | 481.5 | 179 | 24 | 5 | 14 | 9 | + |
| TAG 39:1-12:0 | 696.6 | 479.5 | 179 | 24 | 5 | 14 | 9 | + |
| TAG 40:0-14:0 | 712.6 | 467.5 | 179 | 24 | 5 | 15 | 9 | + |
| TAG 40:1-14:0 | 710.6 | 465.5 | 179 | 24 | 5 | 15 | 9 | + |
| TAG 40:2-14:1 | 710.6 | 463.5 | 179 | 24 | 5 | 15 | 9 | + |
| TAG 40:3-14:1 | 708.6 | 461.5 | 179 | 24 | 5 | 15 | 9 | + |
| TAG 41:0-14:0 | 726.7 | 481.5 | 179 | 24 | 5 | 15 | 9 | + |
| TAG 41:1-14:0 | 724.7 | 479.5 | 179 | 24 | 5 | 15 | 9 | + |
| TAG 41:2-14:0 | 722.7 | 477.5 | 179 | 24 | 5 | 15 | 9 | + |
| TAG 41:3-14:1 | 720.7 | 477.5 | 179 | 24 | 5 | 15 | 9 | + |
| TAG 42:0-14:0 | 740.7 | 495.6 | 179 | 24 | 5 | 15 | 9 | + |
| TAG 42:1-14:0 | 738.7 | 493.6 | 179 | 24 | 5 | 15 | 9 | + |
| TAG 42:2-14:1 | 736.7 | 493.6 | 179 | 24 | 5 | 15 | 9 | + |
| TAG 42:3-14:1 | 734.7 | 491.6 | 179 | 24 | 5 | 15 | 9 | + |
| TAG 43:0-14:1 | 754.7 | 509.4 | 179 | 24 | 5 | 15 | 9 | + |
| TAG 43:1-14:0 | 752.7 | 507.4 | 179 | 24 | 5 | 15 | 9 | + |
| TAG 43:2-14:0 | 750.7 | 505.4 | 179 | 24 | 5 | 15 | 9 | + |
| TAG 43:3-14:0 | 748.7 | 505.4 | 179 | 24 | 5 | 15 | 9 | + |
| TAG 44:0-14:0 | 768.7 | 523.6 | 179 | 24 | 5 | 16 | 9 | + |
| TAG 44:1-14:0 | 766.7 | 521.6 | 179 | 24 | 5 | 16 | 9 | + |
| TAG 44:2-14:0 | 764.7 | 519.6 | 179 | 24 | 5 | 16 | 9 | + |
| TAG 44:3-14:1 | 762.7 | 519.6 | 179 | 24 | 5 | 16 | 9 | + |
| TAG 44:4-16:1 | 760.7 | 489.5 | 179 | 24 | 5 | 16 | 9 | + |
| TAG 45:0-15:0 | 782.5 | 523.6 | 179 | 24 | 5 | 16 | 9 | + |
| TAG 45:0-16:0 | 782.5 | 509.6 | 179 | 24 | 5 | 16 | 9 | + |
| TAG 45:1-16:0 | 782.5 | 507.6 | 179 | 24 | 5 | 16 | 9 | + |
| TAG 45:2-16:1 | 782.5 | 507.6 | 179 | 24 | 5 | 16 | 9 | + |
| TAG 45:3-16:1 | 780.5 | 505.6 | 179 | 24 | 5 | 16 | 9 | + |
| TAG 46:0-14:0 | 796.7 | 551.4 | 179 | 24 | 5 | 18 | 9 | + |
| TAG 46:1-16:1 | 794.7 | 523.4 | 179 | 24 | 5 | 18 | 9 | + |
| TAG 46:2-16:1 | 792.7 | 521.4 | 179 | 24 | 5 | 18 | 9 | + |
| TAG 46:3-16:1 | 790.7 | 519.4 | 179 | 24 | 5 | 18 | 9 | + |
| TAG 46:4-16:1 | 788.7 | 517.4 | 179 | 24 | 5 | 18 | 9 | + |
| TAG 46:5-16:1 | 786.7 | 515.4 | 179 | 24 | 5 | 18 | 9 | + |
| TAG 47:0-16:0 | 810.7 | 537.7 | 179 | 24 | 5 | 18 | 9 | + |
| TAG 47:1-16:0 | 808.7 | 535.7 | 179 | 24 | 5 | 18 | 9 | + |
| TAG 47:2-16:0 | 806.7 | 533.7 | 179 | 24 | 5 | 18 | 9 | + |
| TAG 47:3-16:1 | 804.7 | 533.7 | 179 | 24 | 5 | 18 | 9 | + |
| TAG 48:0-16:0 | 824.6 | 551.3 | 179 | 24 | 5 | 18 | 9 | + |
| TAG 48:1-16:0 | 822.6 | 549.3 | 179 | 24 | 5 | 18 | 9 | + |
| TAG 48:2-16:0 | 820.6 | 547.3 | 179 | 24 | 5 | 18 | 9 | + |
| TAG 48:3-16:1 | 818.6 | 547.3 | 179 | 24 | 5 | 18 | 9 | + |
| TAG 48:4-16:1 | 816.6 | 545.3 | 179 | 24 | 5 | 18 | 9 | + |
| TAG 48:5-16:2 | 814.6 | 545.3 | 179 | 24 | 5 | 18 | 9 | + |
| TAG 48:6-16:2 | 812.6 | 543.3 | 179 | 24 | 5 | 18 | 9 | + |
| TAG 49:0-16:0 | 838.8 | 565.7 | 179 | 24 | 5 | 18 | 9 | + |
| TAG 49:1-16:0 | 836.8 | 563.7 | 179 | 24 | 5 | 18 | 9 | + |
| TAG 49:2-16:0 | 834.8 | 561.7 | 179 | 24 | 5 | 18 | 9 | + |
| TAG 49:3-16:1 | 832.8 | 561.7 | 179 | 24 | 5 | 18 | 9 | + |
| TAG 49:4-16:1 | 830.8 | 559.7 | 179 | 24 | 5 | 18 | 9 | + |
| TAG 50:0-16:0 | 852.6 | 579.3 | 179 | 24 | 5 | 19 | 9 | + |
| TAG 50:1-16:0 | 850.6 | 577.3 | 179 | 24 | 5 | 19 | 9 | + |
| TAG 50:2-16:0 | 848.6 | 575.3 | 179 | 24 | 5 | 19 | 9 | + |
| TAG 50:3-16:1 | 846.6 | 575.3 | 179 | 24 | 5 | 19 | 9 | + |
| TAG 50:4-16:1 | 844.6 | 573.3 | 179 | 24 | 5 | 19 | 9 | + |
| TAG 50:5-18:2 | 842.6 | 545.3 | 179 | 24 | 5 | 19 | 9 | + |
| TAG 50:6-18:2 | 840.6 | 543.3 | 179 | 24 | 5 | 19 | 9 | + |
| TAG 51:0-17:0 | 866.8 | 579.7 | 179 | 24 | 5 | 19 | 9 | + |
| TAG 51:0-18:0 | 866.8 | 565.7 | 179 | 24 | 5 | 19 | 9 | + |
| TAG 51:1-18:1 | 864.8 | 565.7 | 179 | 24 | 5 | 19 | 9 | + |
| TAG 51:2-18:1 | 862.8 | 563.7 | 179 | 24 | 5 | 19 | 9 | + |
| TAG 51:3-18:1 | 860.8 | 561.7 | 179 | 24 | 5 | 19 | 9 | + |
| TAG 51:4-18:2 | 858.8 | 561.7 | 179 | 24 | 5 | 19 | 9 | + |
| TAG 52:0-18:0 | 880.7 | 579.3 | 179 | 24 | 5 | 19 | 9 | + |
| TAG 52:1-18:1 | 878.7 | 579.3 | 179 | 24 | 5 | 19 | 9 | + |
| TAG 52:2-18:1 | 876.7 | 577.3 | 179 | 24 | 5 | 19 | 9 | + |
| TAG 52:3-18:1 | 874.7 | 575.3 | 179 | 24 | 5 | 19 | 9 | + |
| TAG 52:4-18:2 | 872.7 | 575.3 | 179 | 24 | 5 | 19 | 9 | + |
| TAG 52:5-18:2 | 870.6 | 573.3 | 179 | 24 | 5 | 19 | 9 | + |
| TAG 52:6-18:2 | 868.6 | 571.3 | 179 | 24 | 5 | 19 | 9 | + |
| TAG 52:7-18:2 | 866.6 | 569.3 | 179 | 24 | 5 | 19 | 9 | + |
| TAG 53:1-18:1 | 892.7 | 593.3 | 179 | 24 | 5 | 19 | 9 | + |
| TAG 53:2-18:1 | 890.7 | 591.3 | 179 | 24 | 5 | 19 | 9 | + |
| TAG 54:0-18:0 | 908.7 | 607.3 | 179 | 24 | 5 | 19 | 9 | + |
| TAG 54:1-18:1 | 906.7 | 607.3 | 179 | 24 | 5 | 19 | 9 | + |
| TAG 54:2-18:1 | 904.7 | 605.3 | 179 | 24 | 5 | 19 | 9 | + |
| TAG 54:3-18:1 | 902.7 | 603.4 | 179 | 24 | 5 | 19 | 9 | + |
| TAG 54:4-18:1 | 900.6 | 601.4 | 179 | 24 | 5 | 19 | 9 | + |
| TAG 54:5-18:2 | 898.6 | 601.4 | 179 | 24 | 5 | 19 | 9 | + |
| TAG 54:6-18:2 | 896.6 | 599.4 | 179 | 24 | 5 | 19 | 9 | + |
| TAG 54:7-18:2 | 894.6 | 597.4 | 179 | 24 | 5 | 19 | 9 | + |
| TAG 54:8-18:2 | 892.6 | 597.4 | 179 | 24 | 5 | 19 | 9 | + |
| TAG 56:0-20:0 | 936.7 | 607.3 | 179 | 24 | 5 | 20 | 8 | + |
| TAG 56:1-18:1 | 934.7 | 635.3 | 179 | 24 | 5 | 20 | 8 | + |
| TAG 56:2-18:1 | 932.7 | 633.3 | 179 | 24 | 5 | 20 | 8 | + |
| TAG 56:3-18:1 | 930.7 | 631.3 | 179 | 24 | 5 | 20 | 8 | + |
| TAG 56:4-18:2 | 928.7 | 631.3 | 179 | 24 | 5 | 20 | 8 | + |
| TAG 56:5-18:2 | 926.7 | 629.4 | 179 | 24 | 5 | 20 | 8 | + |
| TAG 56:6-20:4 | 924.7 | 603.4 | 179 | 24 | 5 | 20 | 8 | + |
| TAG 57:0-19:0 | 950.9 | 635.7 | 179 | 24 | 5 | 20 | 8 | + |
| TAG 58:0-18:0 | 964.9 | 655.3 | 179 | 24 | 5 | 21.5 | 8 | + |
| TAG 58:1-18:0 | 962.9 | 657.3 | 179 | 24 | 5 | 21.5 | 8 | + |
| TAG 58:2-18:2 | 960.9 | 655.3 | 179 | 24 | 5 | 21.5 | 8 | + |
| TAG 58:3-18:2 | 958.9 | 657.3 | 179 | 24 | 5 | 21.5 | 8 | + |
| TAG 58:4-18:2 | 956.9 | 659.3 | 179 | 24 | 5 | 21.5 | 8 | + |
| TAG 58:5-20:4 | 954.9 | 633.3 | 179 | 24 | 5 | 21.5 | 8 | + |
| TAG 58:6-20:4 | 952.9 | 631.3 | 179 | 24 | 5 | 21.5 | 8 | + |
| TAG 58:7-20:4 | 950.9 | 629.3 | 179 | 24 | 5 | 21.5 | 8 | + |
| TAG 58:8-20:4 | 948.9 | 627.3 | 179 | 24 | 5 | 21.5 | 8 | + |
| TAG 60:0 - 20:0 | 992.9 | 663.8 | 179 | 24 | 5 | 21.5 | 8 | + |
| TAG 60:1 - 20:0 | 990.9 | 661.8 | 179 | 24 | 5 | 21.5 | 8 | + |
| TAG 60:2 - 20:0 | 988.9 | 659.8 | 179 | 24 | 5 | 21.5 | 8 | + |
| TAG 60:3 - 20:0 | 986.9 | 657.8 | 179 | 24 | 5 | 21.5 | 8 | + |
| TAG 60:4 - 20:0 | 984.9 | 655.8 | 179 | 24 | 5 | 21.5 | 8 | + |
